# Transcriptome sequencing of *Saccharina japonica* sporophytes during whole developmental periods reveals regulatory networks underlying alginate and mannitol biosynthesis

**DOI:** 10.1186/s12864-019-6366-x

**Published:** 2019-12-12

**Authors:** Zhanru Shao, Pengyan Zhang, Chang Lu, Shaoxuan Li, Zhihang Chen, Xiuliang Wang, Delin Duan

**Affiliations:** 1 CAS Key Laboratory of Experimental Marine Biology, Center for Ocean Mega-Science, Institute of Oceanology, Chinese Academy of Sciences, Qingdao, 266071 People’s Republic of China; 2 Laboratory for Marine Biology and Biotechnology, Qingdao National Laboratory for Marine Science and Technology, Jimo, Qingdao, 266237 People’s Republic of China; 3 Yellow Sea Fisheries Research Institute, Chinese Academy of Fishery Sciences, Qingdao, 266071 People’s Republic of China; 4 University of the Chinese Academy of Sciences, Beijing, 100093 People’s Republic of China; 5 Qingdao Academy of Agricultural Sciences, Qingdao, 266100 People’s Republic of China; 6 State Key Laboratory of Bioactive Seaweed Substances, Qingdao Brightmoon Seaweed Group Co Ltd, Qingdao, 266400 People’s Republic of China

**Keywords:** Alginate, Mannitol, Transcriptome, Regulatory networks, Growth, Development, *Saccharina japonica*

## Abstract

**Background:**

Alginate is an important cell wall component and mannitol is a soluble storage carbon substance in the brown seaweed *Saccharina japonica*. Their contents vary with kelp developmental periods and harvesting time. Alginate and mannitol regulatory networks and molecular mechanisms are largely unknown.

**Results:**

With WGCNA and trend analysis of 20,940 known genes and 4264 new genes produced from transcriptome sequencing of 30 kelp samples from different stages and tissues, we deduced that ribosomal proteins, light harvesting complex proteins and “*imm* upregulated 3” gene family are closely associated with the meristematic growth and kelp maturity. Moreover, 134 and 6 genes directly involved in the alginate and mannitol metabolism were identified, respectively. *Mannose-6-phosphate isomerase* (*MPI2*), *phosphomannomutase* (*PMM1*), *GDP-mannose 6-dehydrogenase* (*GMD3*) and *mannuronate C5-epimerase* (*MC5E70* and *MC5E122*) are closely related with the high content of alginate in the distal blade. Mannitol accumulation in the basal blade might be ascribed to high expression of *mannitol-1-phosphate dehydrogenase* (*M1PDH1*) and *mannitol-1-phosphatase* (*M1Pase*) (in biosynthesis direction) and low expression of *mannitol-2-dehydrogenase* (*M2DH*) and *Fructokinase* (*FK*) (in degradation direction). Oxidative phosphorylation and photosynthesis provide ATP and NADH for mannitol metabolism whereas glycosylated cycle and tricarboxylic acid (TCA) cycle produce GTP for alginate biosynthesis. RNA/protein synthesis and transportation might affect alginate complex polymerization and secretion processes. Cryptochrome (CRY-DASH), xanthophyll cycle, photosynthesis and carbon fixation influence the production of intermediate metabolite of fructose-6-phosphate, contributing to high content of mannitol in the basal blade.

**Conclusions:**

The network of co-responsive DNA synthesis, repair and proteolysis are presumed to be involved in alginate polymerization and secretion, while upstream light-responsive reactions are important for mannitol accumulation in meristem of kelp. Our transcriptome analysis provides new insights into the transcriptional regulatory networks underlying the biosynthesis of alginate and mannitol during *S. japonica* developments.

## Background

*Saccharina japonica* is an important commercial seaweed in Asia, with industrial cultivation dating from the 1950s and current annual production of over 7.65 million tons wet weight (http://www.fao.org/fishery/species/2776/en) [1]. In addition to being edible, *S. japonica* is widely used as raw material for chemical and pharmaceutical application due to its diverse metabolic compounds such as alginate, fucoidan, mannitol and laminarin [2–5]. Of these compounds, the most abundant metabolites of kelp dry weight are alginate (25%±) and mannitol (15%±) and these compounds are major extracts in the kelp industry, because alginate has valuable gelling, viscosifying and stabilizing properties and mannitol has high osmosis, plasticity and derivatives properties [6]. Unlike land plants and other algal phyla that synthesize cellulose and sucrose, brown algae synthesize alginate as the main component of cell walls and mannitol as the major carbon storage substance [7, 8]. Some studies regarding to variations in alginate and mannitol between months and structures in brown seaweeds have been reported [9–11]. To date, the biosynthesis pathways of alginate and mannitol in algae and regulatory mechanism of their contents remain largely unknown [12]. Hence, it is worth investigating the genes involved in alginate and mannitol pathways through transcriptional profiles.

Genome sequencing of *Ectocarpus siliculosus* provides insights into the origin and evolution of these components and reveals their biosynthetic pathways, which enables the following investigations on the underlying regulatory mechanism in brown algae [7, 8, 13]. Genes encoding mannose-6-phosphate isomerases (MPIs) that catalyze the production of mannose-6-phosphate from fructose-6-phosphate (F6P) have been annotated in *E. siliculosus* and *S. japonica* [7, 14]. However, no mannose-1-phosphate guanylyltransferase (MPG) sequences has yet been annotated from brown algal genomes, and it is believed that MPI can substitute for MPG function [7, 15]. In addition, phosphomannomutase (PMM) is proved to use both mannose-1-phosphate and glucose-1-phosphate as substrates [16]. GDP-mannose 6-dehydrogenase (GMD) isolated from *E. siliculosus* uses GDP-mannose as the only substrate to catalyze the conversion to GDP-mannuronic acid [17]. Previously, we have experimentally validated two GMDs from *S. japonica* in response to heat and desiccation stresses [18]. For the last step of alginate biosynthesis, abundant mannuronate C5-epimerase (MC5E) sequences are available with only two recombinant MC5Es being characterized [19, 20]. Generally, there are four steps in the mannitol metabolic pathway: 1) mannitol-1-phosphate dehydrogenase (M1PDH) reduces F6P to mannitol-1-phosphate (M1P); 2) mannitol-1-phosphatase (M1Pase) hydrolyzes M1P to mannitol; 3) mannitol-2-dehydrogenase (M2DH) oxidizes mannitol to fructose; and 4) fructokinase catalyzes the production of F6P from fructose [13, 21]. In the brown algal mannitol pathway, M1PDH was the first enzyme heterologously over-expressed in *Escherichia coli* [22]. Subsequently, *M1Pase* from *E. siliculosus* was confirmed to hydrolyze M1P to mannitol [23, 24]. Moreover, our previous study proved that M2DH from *S. japonica* is highly active in the reduction reaction of fructose to mannitol [25]. These previous studies mainly focused on the characterization of individual enzyme from both pathways. Nevertheless, the regulatory networks underlying alginate and mannitol biosynthesis is not clear yet.

In this study, transcriptomic data mining via profile analysis and weighted gene co-expression network analysis (WGCNA) identified gene families correlated with development, modules correlated with traits and potential hub genes responsible for alginate and mannitol biosynthesis in *Saccharina*. Our study paves the way for elucidating the regulatory mechanism in alginate and mannitol biosynthesis, and sheds lights on the genetic adaption of kelp under increasing light and temperature conditions with developments.

## Results

### Sequencing data interpretation

Totally, 30 kelp samples were subjected to transcriptome sequencing and data analysis (Table 1; Additional file 1: Figure S1). All the RNA integrity numbers (RINs) were between 7.2–9.2, which showed that all the samples were qualified for deep sequencing (Additional file 2: Table S1). High-throughput sequencing generated 45.69–84.83 million of 150-bp paired-end reads in each library (Table 2). After data filtering, 1,751,262,386 high quality reads were produced (98.20% of clean reads) with an average Q30% > 96.00%. rRNA removed reads were mapped with our previous completed *S. japonica* genome (NCBI: MEHQ00000000) with a mapping ratio of c. 80%, except for Ap1–3 sample which had a rather low ratio of 50.46% (Table 2; Additional file 2: Table S1). Assembled transcriptomes were annotated according to 24,419 reference genes. In total, 20,940 known genes (ID starts with “GENE_”) and 4264 novel genes (ID starts with “XLOC_”) were obtained. There are 1957 novel genes showing high identities with sequences from *E. siliculosus*, among which 34.3% were annotated as unknown or hypothetical proteins. KEGG pathway annotation showed two main categories of “Metabolism” and “Genetic information processing” (Additional file 3: Figure S2). Statistics of transcriptomes sequencing output are in Additional file 2: Table S1.

**Table 1:** The collection background of *S. japonica* samples

Collection date	Sample ID	Tissue site	Replicates	Seawater temperature
22nd January	JaB	Basal	JaB-1, JaB-2, JaB-3	5.2 °C
4th March	MhB	Basal	MhB-1, MhB-2, MhB-3	4.6 °C
10th April	ApB	Basal	ApB-1, ApB-2, ApB-3	5.6 °C
	Ap1	1/3	Ap1–1, Ap1–2, Ap1–3	
	Ap2	2/3	Ap2–1, Ap2–2, Ap2–3	
	ApD	Distal	ApD-1, ApD-2, ApD-3	
10th May	MyB	Basal	MyB-1, MyB-2, MyB-3	9.0 °C
	MyD	Distal	MyD-1, MyD-2, MyD-3	
16th June	JuB	Basal	JuB-1, JuB-2, JuB-3	13.2 °C
	JuD	Distal	JuD-1, JuD-2, JuD-3

**Table 2:** The output and quality control of the RNA-Seq data

	Maximum	Minimum	Average
Clean data (bp)	12,724,976,400	6,852,831,900	8,916,922,600
HQ clean data (bp)	12,214,001,205	6,606,166,213	8,595,871,647
Q30 (%)	96.69%	95.05%	96.00%
GC (%)	57.26%	55.72%	56.52%
Clean reads No.	84,833,176	45,685,546	594,461,501
HQ clean reads No.	82,982,428	44,857,256	58,375,413
% HQ clean reads	98.47%	97.82%	98.21%
Mapped reads No.	65,017,939	32,881,766	45,563,696
Mapping ratio	82.76%	50.46%	80.33%
Total genes	25,204		

### Trend analysis and functional enrichment of differentially expressed genes (DEGs)

DEGs in different kelp developmental periods and tissue portions were clustered into 29 and 25 profiles, respectively (Additional file 4: Figure. S3). We selected two representative profiles: profile29 (985 genes) with increasing DEGs expression and profile0 (1319 genes) with decreasing trend from January to June. Twenty DEGs encoding ribosomal proteins (RPs) were enriched in the profile29 (Q < 0.05) (Additional file 5: Table S2). “Oxidative phosphorylation”, “photosynthesis-antenna proteins”, “photosynthesis and carbon fixation pathways” were enriched in profile0, with a decreasing trend from juvenile to mature sporophytes (Q < 0.05) (Additional file 5: Table S2). While for DEGs in different tissues, we selected 3 profiles with increase pattern [profiles 25 (367 genes), 22 (322 genes) and 16 (303 genes)] and 2 with decrease pattern [profiles 9 (1619 genes) and 0 (1359 genes)] (Additional file 5: Table S2). DEGs in “ABC transporters and RNA transport pathways” were highly enriched (*p* < 0.05) and gradually increased from basal to distal blade. “Polyunsaturated fatty acids (e.g. arachidonic acid and linoleic acid) metabolisms” were also enriched in this pattern (Q < 0.05). Profile9 and profile0 enriched “photosynthesis-antenna protein”, “secondary metabolites biosynthesis and thiamine (V_B1_) metabolism”, and complex metabolic pathways known as “microbial metabolism in diverse environments” under the KEGG nomenclature (Q < 0.05). Notably, “*imm* upregulated 3” gene family were highly expressed in juvenile sporophytes, with 28 genes enriched in profile0 (Additional file 6: Table S3). In the basal blade, 32 “*imm* upregulated 3” genes were enriched (Additional file 6: Table S3). We analyzed the expression profiles of genes related with energy-producing metabolisms (Table 3). Although there is no obvious expression tendency from January to June, genes encoding key enzymes in TCA cycle and glyoxylate cycle were highly expressed in the distal blade of kelp, whereas those crucial genes in glycolysis, gluconeogenesis and oxidative phosphorylation were highly expressed in basal blade compared with distal blade.

**Table 3:** The expression levels of key enzymes involved in energy-producing metabolisms between basal and distal blade of kelp

Pathway	Rate-limiting enzyme	Gene ID	Fold change		
			ApD vs ApB	MyD vs MyB	JuD vs JuB
TCA cycle	Citrate synthase	GENE_007839	−2.29	−2.11	−11.25
	Oxoglutarate dehydrogense	GENE_002415	−1.78	−2.93	−3.95
		GENE_002283	−1.75	−1.36	− 1.86
		XLOC_013477	1.38	−1.73	−2.86
Glyoxylate cycle	Isocitrate lyase	GENE_025653	−7.81	−46.48	−19.09
	Malate synthase	GENE_024070	−2.58	−9.05	−14.19
Glycolysis	Glucokinase	GENE_019969	3.24	12.18	7.35
		GENE_027705	7.38	9.60	5.18
	Pyrophosphate- phosphofructose kinase	GENE_017153	7.63	13.50	5.19
		XLOC_033414	3.65	6.02	4.30
		GENE_023282	1.18	1.34	0.84
	Pyruvate kinase	GENE_006234	1.13	1.63	1.68
Gluconeogenesis	Fructose-1,6-bisphosphatase	GENE_010339	2.90	2.40	2.85
		GENE_004642	1.60	1.14	1.27
	Phosphoenolpyruvate carboxykinase (ATP)	XLOC_018117	46.29	38.48	18.73
		GENE_015129	28.30	30.78	22.01
Oxidative phosphorylation	ATP synthase	GENE_008643	1.75	2.32	0.69
		GENE_004216	4.77	9.00	3.29
		GENE_020559	1.85	2.24	0.94
		GENE_025468	2.63	2.36	1.47
		GENE_024356	3.00	1.44	0.57

### Alginate and mannitol content variations during the kelp developmental periods

**Fig. 1:**
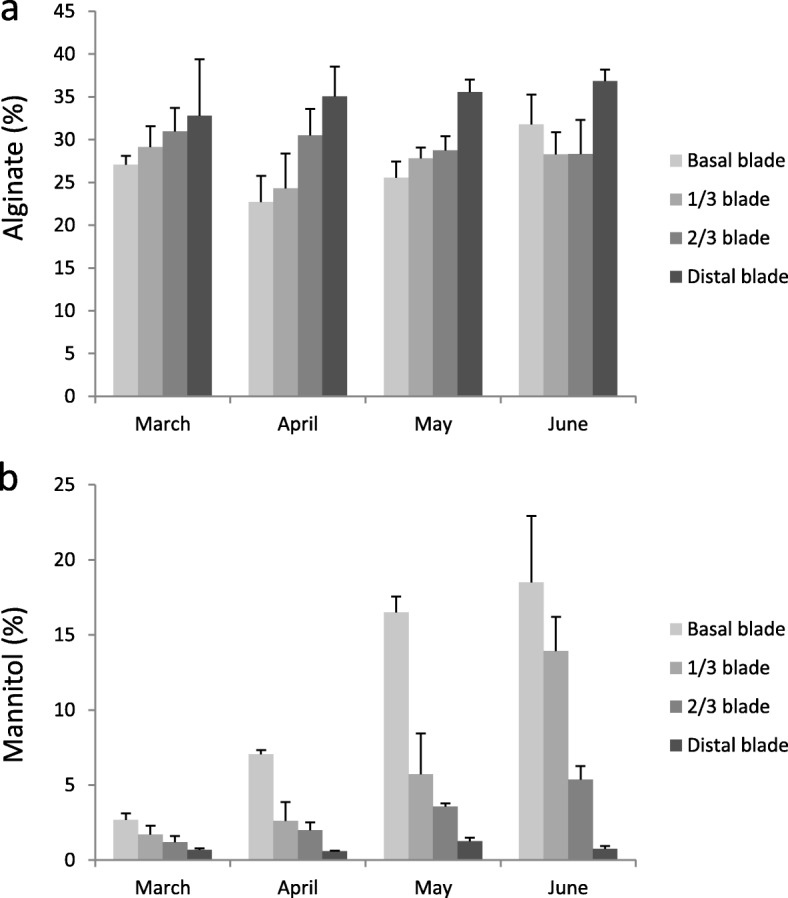
Contents of alginate and mannitol detected in *S. japonica* samples collected from different developmental stages and tissue parts. **a** Content variations of alginate from March to June. **b** Content variations of mannitol from March to June

Mannitol and alginate contents in *S. japonica* were detected at different periods from January to June (Fig. 1; Additional file 7: Figure S4). Average content of alginate in individual kelp collected from each month was c. 30% without significant difference from month to month. However, mannitol content gradually increased from 1.56% (in March) to 9.63% (in June), a 6.18-fold difference between the mature and the juvenile sporophytes (Fig. 1). The contents of these two metabolites varied in different tissue portions. For instance in April, alginate content was up-regulated from basal blade (22.71%) to distal blade (35.06%) whereas mannitol was down-regulated from 7.04 to 0.58% (Fig. 1; Additional file 7: Figure S4). Alginate content was constantly higher in the distal blade than in the basal blade (1.16–1.54-fold change). On the contrary, the basal blade contained more mannitol than the distal blade, especially in mature sporophytes in June (25.04-fold). In general, mannitol was up-regulated from juvenile to mature sporophytes and the variations of mannitol and alginate showed opposite patterns from basal to distal blade (Fig. 1).

### Identification of alginate/mannitol-related genes and their transcriptional profiles

**Fig. 2:**
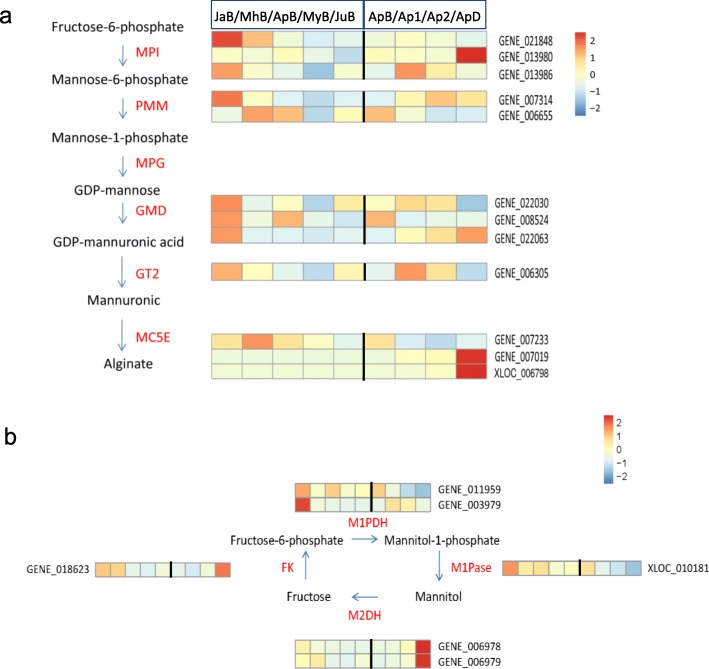
Transcriptional patterns of the genes involved in alginate and mannitol metabolism. **a** Expression levels of alginate biosynthetic genes. **b** Expression levels of mannitol metabolic genes. The order of each row is: JaB, MhB, ApB, MyB, JuB, ApB, Ap1, Ap2, ApD. MPI1: GENE_021848; MPI2: GENE_013980; MPI3: GENE_013986; PMM1: GENE_007314; PMM2: GENE_006655; GMD1: GENE_022030; GMD2: GENE_008524; GMD3: GENE_022063; GT2: GENE_006305; MC5E1: GENE_007233; MC5E70: GENE_007019; MC5E122: XLOC_006798; M1PDH1: GENE_011959; M1PDH2: GENE_003979; M1Pase: XLOC_010181; M2DH1: GENE_006978; M2DH2: GENE_006979; FK: GENE_018623 *MPI* Mannose-6-phosphate isomerase, *PMM* Phosphomannomutase, *GMD* GDP-mannose 6-dehydrogenase, *GT2* Beta-1,3-glucan synthases (family GT2), *MC5E* Mannuronate C5-epimerase, *M1PDH* Mannitol-1-phosphate dehydrogenase, *M1Pase* Mannitol-1-phosphatase, *M2DH* Mannitol-2-dehydrogenase, *FK* Fructokinase

Based on the annotation of our previously sequenced *S. japonica* genome, we screened 134 genes encoding enzymes catalyzing alginate biosynthesis, including 3 *MPI*, 2 *PMM*, 3 *GMD*, 1 *GT2* and 125 *MC5E* genes. We identified 6 genes encoding enzymes involved in the mannitol metabolism, of which 3 sequences (*M1PDH* and *M1Pase*) were for mannitol biosynthesis and the rest 3 sequences (*M2DH* and *FK*) were for degradation (Additional file 8: Table S4). Transcriptional profiles of these genes from RNA-Seq data are shown in Fig. 2. Transcripts of *MPI1* (GENE_021848), *MPI3* (GENE_013986), *PMM1* (GENE_007314) and *GMD3* (GENE_022063) were down-regulated from January to June, whereas other genes did not exhibit specific transcriptional pattern, especially for *MC5E* gene family which contains 125 genes with very diverse transcriptional profiles. Figure 2a shows the expression levels of 3 representative *MC5Es*, among which MC5E70 (GENE_007019) and MC5E122 (XLOC_006798) were highly expressed in the distal blade. *PMM2* (GENE_006655) and *GMD3*(GENE_022063) exhibited opposite expression patterns: *PMM2* decreased and *GMD3* increased from basal blade to distal blade (Fig. 2a). *M1PDH1* (GENE_003979) and *M1Pase* (XLOC_010181) expression were gradually down-regulated and *M2DH* (GENE_006978 and GENE_006979) and *FK* (GENE_018623) were remarkably up-regulated from basal to distal blade. Expression levels of all the 6 genes in mannitol cycle were higher in juvenile sporophytes than in later stages (Fig. 2b).

### Identification of alginate−/mannitol- co-expressed genes and pathways via module-trait correlations

**Fig. 3:**
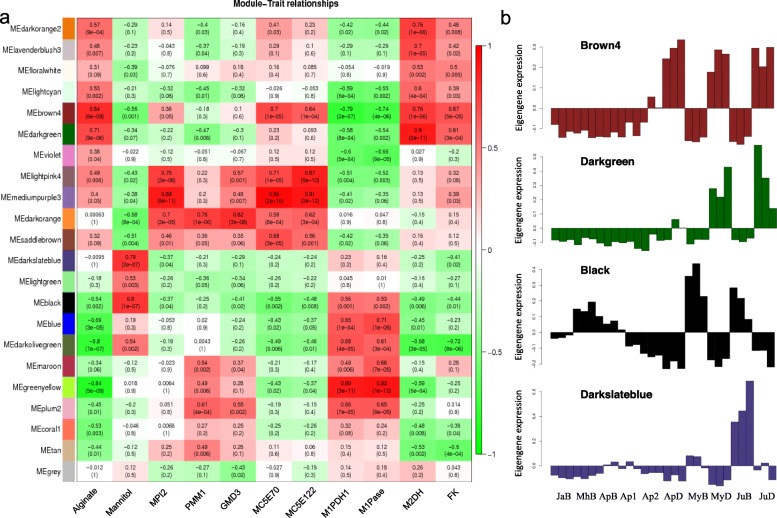
Module-trait correlations and gene expression patterns of the top 2 modules correlated with alginate and mannitol contents. **a** Module-trait relationships and corresponding *p* values. The color scale on the right shows correlations from − 1 (green) to 1 (red). Panels on the left represent alginate and mannitol content as traits. Other panels show the expression variations of each biosynthetic gene as a trait. **b** Expression patterns of each selected module. “Brown4” and “Darkgreen” were highly correlated with alginate content, whereas “Black” and “Darkslateblue” were highly correlated with mannitol content

WGCNA analysis resulted in 22 distinct modules. Additional file 9: Figure S5 shows the hierarchical cluster tree for modules of co-expressed genes with each branch constituting a module and each leaf as one gene. Additional file 10: Table S5 lists the number of genes clustered in each module. Alginate and mannitol module-trait correlation analysis was conducted based on WGCNA data. Figure 3a shows correlations from − 1 (green) to 1 (red), which revealed that the “brown4” module was closely related to alginate content (r = 0.84, *p* = 6 × 10^− 9^) and “black” module was highly correlated to mannitol (r = 0.80, *p* = 1 × 10^− 7^). Figure 3a shows the opposite module-trait correlation pattern between alginate and mannitol biosynthesis: positive correlated modules for alginate concentrated mannitol-negatively correlated modules and vice versa. Figure 3b shows the top 2 modules for each correlation analysis: “brown4” and “darkgreen” were correlated with alginate whereas “black” and “darkslateblue” were correlated with mannitol.

Although the “brown4” and “black” modules were highly correlated with alginate and mannitol contents, none of their biosynthetic genes were found in these two modules (Additional file 8: Table S4). Genes involved in alginate biosynthesis: *MPI2* (GENE_013980), *PMM1* (GENE_007314), *GMD3* (GENE_022063), *MC5E70* (GENE_007019) and *MC5E122* (XLOC_006798) appeared in “darkorange” and “mediumpurple3” modules (Fig. 3a). The two modules clustered genes with higher expression levels in the distal blade than those in the basal blade of kelp, as the “brown4” module (Additional file 11: Figure S6a, b). *M1PDH1* and *M1Pase* (for mannitol synthesis) fell into the module of “greenyellow” (Fig. 3a), in which gene expression levels were relatively higher in basal blade (Fig. 3b; Additional file 11: Figure S6c). However, their transcripts were highly up-regulated in juvenile sporophytes, but mannitol content was higher in adult kelp. *M2DH* and *FK* (for mannitol degradation) fell into “darkgreen” and “bown4” modules (Fig. 3a) which showed the opposite expression patterns with *M1PDH1* and *M1Pase* (for mannitol synthesis).

The “brown4” and “darkgreen” modules indicated that the expression levels of those genes correlated with alginate biosynthesis were constantly lower in the basal samples from January to June (JaB, MhB, ApB, MyB toJuB), but were higher in the distal blade (ApD, MyD and JuD) (Fig. 3b). The enriched pathways in “brown4” module included TCA cycle, carbon metabolism and amino acid metabolism etc. (Additional file 12: Table S6). In the “darkgreen” module, pathways were concentrated on DNA and protein regulation, including DNA replication (7 DEGs), pyrimidine metabolism (12 DEGs), nucleotide excision repair (7 DEGs), and ubiquitin mediated proteolysis (11 DEG) (Additional file 12: Table S6). Seven representative genes were summarized in Table 4.

**Table 4:** The description of representative genes in DNA and protein regulation pathways which are highly correlated with “darkgreen” module (*p* < 0.05)

Pathway	Gene ID	Connectivity	Annotation
DNA replication	GENE_017950	216.81	Cdc21-like protein
	GENE_029087	94.91	DNA polymerase
Pyrimidine metabolism	GENE_012005	112.52	CTP synthase
	XLOC_025500	119.87	RNA polymerase II
Nucleotide excision repair	XLOC_012937	146.32	Transcription factor II H
Ubiquitin mediated proteolysis	XLOC_014294	71.42	Ubiquitin-conjugating e2 j1
	GENE_001248	84.28	Ubiquitin-conjugating enzyme 1

Genes correlated with mannitol content were up-regulated with the kelp growth and developments (“darkslateblue” module). The expression levels of these genes were higher in the kelp basal blade, with an opposite pattern compared with genes correlated with alginate content (Fig. 3b). Interestingly, “greenyellow” module (*M1PDH*- and *M1Pase*-correlated module in Fig. 3a) enriched pathways of energy generation, photosynthesis and photomorphogenesis (*p* < 0.05) (Table 5). We listed all the genes in these pathways (Additional file 13: Table S7) and found that: 1) twenty-two genes were annotated in the most significantly enriched pathway “oxidative phosphorylation”; 2) more than half of the annotated light harvesting complex protein (LHC) genes fell into “greenyellow” module, together with the genes in photosynthesis e.g. PSII, cytochrome b6/f complex, electron transport and ATPase; 3) the complete biosynthetic pathway from ζ-carotene to violaxanthin, the precursor of fucoxanthin, was annotated; 4) one *CRY-DASH* gene which encodes cryptochrome in response to blue light was annotated; and 5) the carbon fixation pathway from ribose-5-phosphate to F6P was significantly enriched with at least 7 genes annotated.

**Table 5:** Genes description in enriched pathways from “greenyellow” module (*p* < 0.05)

Pathway	DEGs in pathway	Gene ID	Annotation
Oxidative phosphorylation	22 (6.23%)	GENE_024356	ATP synthase gamma chain
		GENE_019595	NADH dehydrogenase subunit 10
Photosynthesis - antenna proteins	18 (5.1%)	GENE_022269	Light harvesting protein lhcf6
Photosynthesis	9 (2.55%)	GENE_014910	Photosystem II 12 kDa extrinsic protein
Circadian rhythm - plant	5 (1.42%)	GENE_003847	Cryptochrome 3
		GENE_017333	Phytochrome-like protein 3
Carotenoid biosynthesis	7 (1.98%)	GENE_000415	Zeta-carotene desaturase
		GENE_021497	Lycopene beta cyclase
		GENE_027759	Cytochrome P450
		GENE_017807	Violaxanthin de-epoxidase
		GENE_006795	Flavoprotein Monooxygenase
Carbon fixation in photosynthetic organisms	12 (3.4%)	GENE_000154	Phosphoglycerate kinase
		GENE_008411	Fructose-bisphosphatase
		GENE_002455	Malate dehydrogenase

### Validation of the expression of representative alginate/mannitol-related genes

**Fig. 4:**
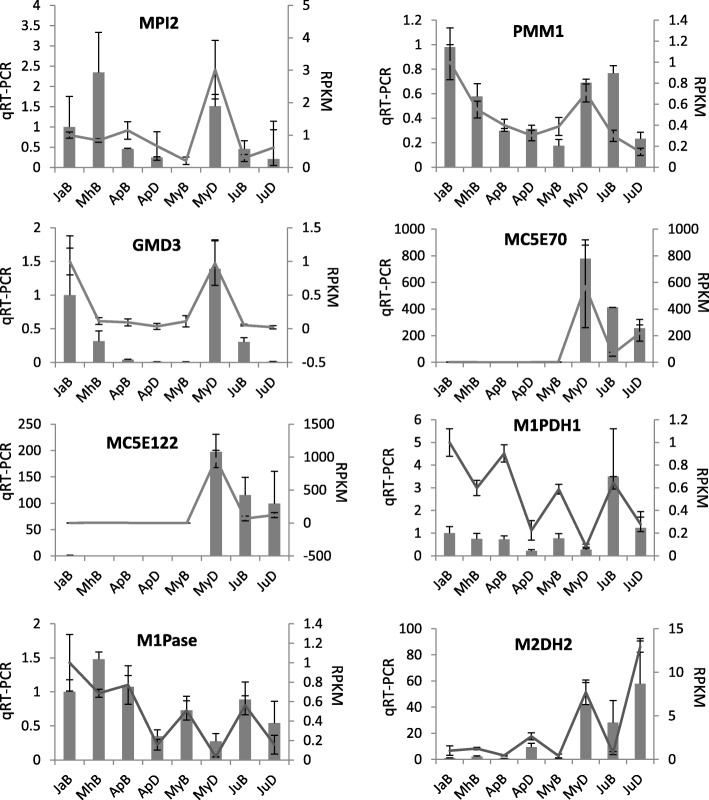
Expression levels of the genes involved in alginate and mannitol metabolism by RNA-Seq and real-time qPCR validation. The histogram shows the RT-qPCR results and the curve indicates the expression levels from RNA-Seq results

Five genes (*MPI2*, *PMM1*, *GMD3*, *MC5E70* and *MC5E122*) correlated with alginate content, and 3 genes (*M1PDH1*, *M1Pase*, *M2DH*) correlated with mannitol were selected for verification with real-time quantitative PCR (RT-qPCR) assay. Their expression patterns detected with RNA-Seq and RT-qPCR were greatly consistent (Fig. 4), indicating the reliability of high-throughput transcriptomes sequencing. *M1PDH1* expression levels were much higher in the adult sporophytes in June than that in earlier developmental stages. It was conflicting with the RNA-Seq but consistent with the extensive accumulation of mannitol in late developmental stages.

### Screening of transcription factors (TFs) from co-expression analysis

Totally, 57 TFs were identified with the online prediction tool PlantTFDB, and their connectivities range from 2.56 to 238.42. All these TF gene sequences were listed in Additional file 14: Table S8. Eight genes encoding heat shock transcription factors (*HSFs*) were distributed in 7 modules, being the most abundant TFs. GENE_023741 in “darkolivegreen” was the *HSF* with highest connectivity and expression levels, which is presumed to have central regulating function. Three *MYB* or MYB DNA binding protein transcription factors and 3 histone-like TFs (*NFY2*, *NFYB3* and *NFYC4*) were identified, among which *NFY2* (GENE_001494 in “darkolivegreen”) and *NFYB3* (GENE_001506 in “darkolivegreen”) exhibited high expression levels and high connectivities. We searched TFs in 4 modules correlated with mannitol and alginate biosynthesis (Table 6; Additional file 15: Table S9). In total, 15 TFs were screened in “black” and “greenyellow” modules, of which 3 *HSFs* (*HSF1*, *HSF3* and *HSFA4C*), 1 *MYB3R* and 1 *E2F* were identified. Only 4 TFs were found in alginate-correlated “brown4” and “darkorange” modules, of which one RNA polymerase and one histone-like TF were identified (Table 6; Additional file 15: Table S9).

**Table 6:** The identified transcription factors in the mannitol/alginate-correlated modules

Gene ID	Name	Module	Pfam domain	Annotation
GENE_023779	HSF1	Greenyellow	HSF	Heat Shock transcription factor (*E. siliculosus*)
GENE_025481	MYB3R	Black	SANT	myb transcription factor (*Nannochloropsis gaditana*)
GENE_008289	E2F	Black	E2F_CC-MB	transcription factor E2F (*E. siliculosus*)
XLOC_031444	Chrac1	Darkorange	CBFD_NFYB_HMF	histone-like transcription factor family (CBF/NF-Y) (*E. siliculosus*)
GENE_024306	rpa12	Darkorange	ZnF_C2C2	RNA polymerase I transcription factor TFIIS subunit RPA12 (*K. flaccidum*)

### Protein interaction networks predicted for mannitol and alginate biosynthesis

**Fig. 5:**
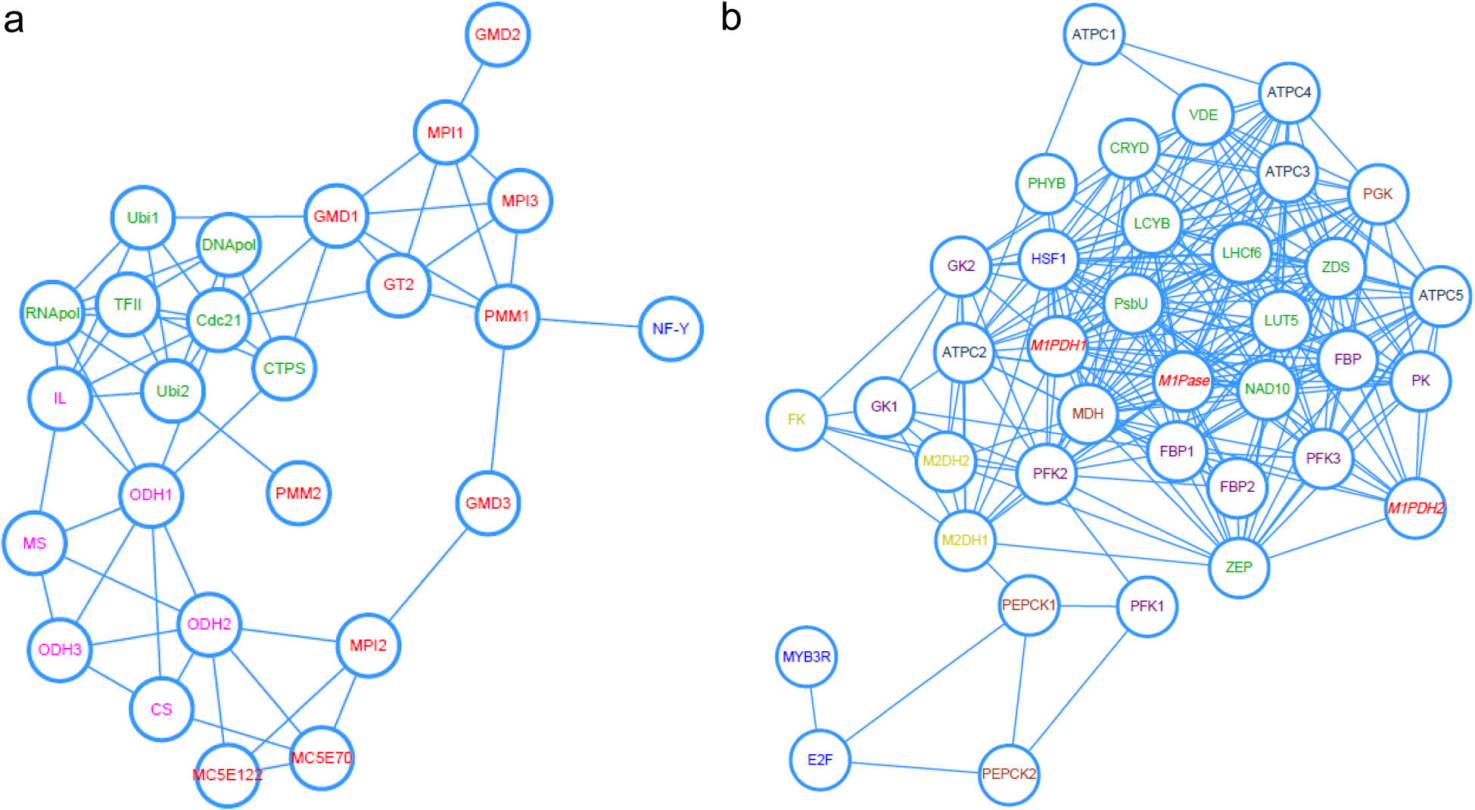
Cytoscape representation of co-expressed genes with alginate and mannitol metabolisms selected by Pearson correlation coefficient ≥ 0.6 or ≤ − 0.6. **a** Protein interaction network for alginate biosynthesis. Genes involved in alginate pathway (red), DNA and protein regulation pathways (green), energy-producing metabolisms (purple) and transcription factor (blue) were indicated. **b** Protein interaction network for mannitol metabolism. Genes involved in mannitol biosynthesis (red), mannitol degradation (yellow), light-responsive reactions (green), energy-producing metabolisms (purple), carbon fixation (brown) and transcription factor (blue) were indicated. Full names for all genes were listed in Abbreviations

We screened the co-expressed genes with alginate and mannitol metabolisms by pearson correlation coefficient ≥ 0.6 or ≤ − 0.6. The cytoscape representation is shown in Fig. 5. Alginate-biosynthetic genes highly interacted with genes in DNA and protein regulation. RNA polymerase II (RNApol) and oxoglutarate dehydrogense (ODH) were hub genes affecting alginate content (Fig. 5a). Mannitol-biosynthetic genes were highly correlated with light-responsive reactions. Light harvesting complex protein (LHC), malate dehydrogenase (MDH) and HSF1 play the most important role in the mannitol metabolism (Fig. 5b).

## Discussion

### Content variations of alginate and mannitol

Alginate and mannitol accumulate in late developmental periods from April to June detected in our study. This is mainly ascribed to the growth characteristic of annual cultivars of *S. japonica*, which experiences a restricted growth from late April, leading to less protein synthesis but more photosynthetic products [26]. *S. japonica* is cultivated upside down, attaching to the cultivation ropes floating on the sea. This hanging mode endows the holdfast and meristem more light, which could explain why mannitol is highly accumulated in the basal blade. On the contrary, alginate content is higher in the distal blade which is in accordance with results by Ji et al. (1984), indicating that this cell wall structure component tends to accumulate in old tissues of *S. japonica* [27]. This phenomenon is consistent with the higher content of cell wall polysaccharides in overwintered blade (old tissues) than that in new tissues of *Laminaira setchellii* [28]. In addition, distal blades are more sensitive to environmental stresses and pathogens [29, 30], and we presume that the increase of alginate in the blade tip might help to resist abiotic stress and pathogens. This deduction has been proposed for stress resistance function of fucoidan [31], another cell wall polysaccharide found in brown algae.

### Transcriptional profiles of genes involved in alginate and mannitol biosynthesis

Alginate biosynthetic genes are more expressed in juvenile sporophytes, which might be ascribed to active cell division in *Saccharina* early developmental periods. Mannuronate C5-epimerase (MC5E) genes show remarkable gene expansion and complex transcriptional profiles, implying their significant role in determining alginate content and structure with kelp developments. This phenomenon was also reported by Ye et al. (2015) [14]. The higher expression levels of *M1PDH1* and *M1Pase* and lower expression of *M2DH* and *FK* in the basal blade were considered to contribute to the higher mannitol content on the mRNA level. Tonon et al. (2017) found that M1PDH and M1Pase are more widespread and diverse than thought [5]. Considering our results, M1PDH and M1Pase should be regarded as the most important enzymes responsible for *Saccharina* mannitol biosynthesis.

### Alginate- and mannitol- correlated pathways responsible for their accumulation

Moradali et al. (2018) reported that F6P is produced via pyruvate metabolism, TCA cycle and gluconeogenesis in bacterial alginate biosynthesis [12]. Our transcriptome analysis identified the importance of glyoxylate cycle in addition to TCA cycle, which might supply GTP for alginate synthesis in brown algae. Except for biosynthesis, alginate has complicated modification and secretion mechanism, which is rarely investigated [32, 33]. In this study, we have identified many DEGs involved in DNA replication, DNA excision repair and ubiquitin mediated proteolysis, which are presumed to function in alginate polymerization and secretion.

Glycolysis, gluconeogenesis and oxidative phosphorylation are correlated with mannitol content, which are presumed to supply ATG and NADH for mannitol metabolism. Photosynthesis and carbon fixation are crucial in providing light energy and intermediate metabolites for mannitol biosynthesis, especially in basal blade. This correlation agrees with the fact that mannitol is the primary product of photosynthesis in kelp [34]. Gravot et al. (2010) reported that *E. siliculosus* accumulated more mannitol under light [35]. In our study, transcripts of light-responsive elements such as cryptochrome, light harvesting proteins and xanthophyll cycle are correlated with mannitol biosynthesis. We presume that these components constitute a continuous set to regulate complex light conditions for the upside down cultivation of kelp and contribute to mannitol accumulation in basal blade.

### TFs related with alginate and mannitol biosynthesis

Transcription factors (TFs) provide a complex control mechanism for modulating plant developmental processes. In mannitol-correlated modules (“black” and “greenyellow”), MYB3R and E2F are considered to contribute predominantly in meristematic cell cycle. It has been reported in *Arabidopsis* that MYB3R functions in cell cycle regulation by activating cytokinesis-related genes [36, 37]. Likewise, E2F was verified to control S phase and cell cycle progression [38, 39]. We thus deduce that the up-regulation of *MYB3R* and *E2F* in the basal blade leads to more active cell division and might indirectly facilitating carbon uptake and storage. Another meristem-enriched transcription factor is HSF, which could function in response to comparatively high temperature around basal blades. *NF-Y* and *TFIIS* genes are present in alginate-correlated “darkorange” module, with transcriptional levels gradually increasing from the basal to the distal blade. According to previous reports, NF-Y was reported to participate in root growth, photosynthesis and stress response whereas TFIIS is related with Pol II binding and complements Pol II active site [40–43]. From our results, we deduced that NF-Y and TFIIS might be important upstream regulatory elements for alginate biosynthesis in *S. japonica*.

### Genes function in basal development and kelp maturity

Genes encoding ribosomal proteins (RPs) are highly up-regulated in the basal blade of kelp, especially in the mature sporophytes, which implies their importance in meristematic development and kelp maturity. In *Arabidopsis*, ribosomal protein (*RPS13*) controls cell elongation and cell division [44]. *RPS10B* affects shoot meristem by promoting axillary shoot development [45]. However, the regulatory mechanism of RPs in algae are not yet investigated to our knowledge, except for RPs in *Porphyra purpurea* that are responsible for blade and conchocelis differentiation [46]. Moreover, RP transcripts are correlated with mannitol content variations, which indicates their potential regulation role in storage carbon biosynthesis in kelp.

Genes encoding light harvesting complex proteins (LHCs) were highly transcribed in the juvenile and basal blades, which indicates that LHCs may compensate for the lower light intensity in winter (juvenile sporophytes) and resist relatively high light intensity in the basal blade. This is consistent with studies of microalgae and other seaweeds that LHCs play an important role in algal developments via adapting to changeable light irradiance [47, 48]. In *E. siliculosus*, many LHC transcripts were down-regulated in *imm* mutant partheno-sporophyte [49]. *Imm* was reported to be a regulatory locus, partially controlling the sporophyte-specific developmental program [14, 49]. In this study, “*imm* upregulated 3” gene family were highly expressed in juvenile and basal blades, indicating that they might have synergistic effects with LHCs for the development of meristem.

## Conclusions

**Fig. 6:**
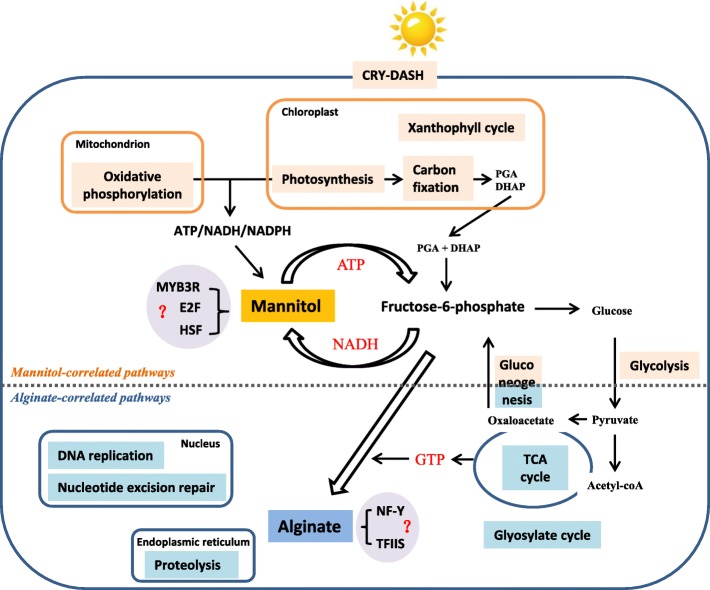
Schematic representation of putative transcriptional regulatory model for alginate and mannitol metabolism in *S. japonica*. Pathways correlated with mannitol metabolism are colored in light orange, with involvement of photosynthesis, oxidative phosphorylation, carbon fixation, glycolysis and gluconeogenesis. Pathways correlated with alginate biosynthesis are colored in cyan, containing glyoxylate cycle, TCA cycle, gluconeogenesis, and RNA/protein synthesis and transportation. The predicted transcription factors are colored in lilac

One transcriptional regulatory network is deduced which integrates responsive reactions correlated with alginate and mannitol biosynthesis (Fig. 6). CRY-DASH, xanthophyll cycle, photosynthesis and carbon fixation might influence the production of intermediate metabolite of F6P, contributing to high content of mannitol in the basal blade. Oxidative phosphorylation and photosynthesis provide ATP and NADH for mannitol metabolism whereas glycosylated cycle and TCA cycle produce GTP for alginate biosynthesis. RNA/protein synthesis and transportation might affect alginate complex polymerization and secretion processes. Our results provide understanding for molecular mechanisms underlying adaption of *Saccharina* to the upside down cultivation and pave a way for functional verification of correlated elements in future.

## Methods

### Kelp sample collections and treatments

Fresh sporophytes of *S. japonica* strain “Zhongke 2” were collected on January 22, March 4, April 10, May 10, and June 16 of 2014 from cultivation rafts in Gaolv Aquaculture Co. Ltd., Rongcheng, Shandong, China (122.6°E, 37.2°N) (Table 1). Samples were washed twice with filtrated seawater and clipped at four tissue sites: basal blade (meristematic region), 1/3 blade, 2/3 blade and distal blade (Additional file 1: Figure S1). Table 1 lists the samples information, with IDs of JaB (basal blade collected in January), MhB (basal blade of March), ApB (basal blade of April), Ap1 (1/3 blade of April), Ap2 (2/3 blade of April), ApD (distal blade of April), MyB (basal blade of May), MyD (distal blade of May), JuB (basal blade of June) and JuD (distal blade of June). Clipped kelpwas dimidiated, with one part frozen in liquid nitrogen for the following RNA isolation and the other desiccated with silica gel for theextraction of alginate and mannitol.

### RNA extraction, library construction and RNA-Seq analysis

Total RNA was extracted using RNApure Plant Kit (ComWin Biotech, Beijing, China). Yield was detected by the NanoDrop 2000 UV-Vis spectrophotometer (Thermo Scientific, Waltham, USA). RNA integrity number (RIN) was detected on Agilent 2100 Bioanalyzer (Agilent Technologies, Santa Clara, USA). Those RNA with RIN > 7.0 were adopted for deep sequencing. The mRNA was enriched with Oligo (dT) beads, cleaved into small pieces with fragmentation buffer and reverse transcribed into cDNA with random primers. Second-strand cDNA was generated using RNase H and DNA polymerase I. cDNA fragments were purified with QiaQuick PCR extraction kit (Qiagen, Duesseldorf, Germany), end repaired, poly(A) added and ligated to adaptors. After PCR amplification, cDNA libraries were paired-end sequenced on Illumina HiSeq™ 2500 platform (San Diego, CA, USA) by Gene Denovo Biotechnology Co. (Guangzhou, China).

### Sequencing data processing and interpretation

Raw reads containing adapters, > 10% of unknown nucleotides and > 50% of base quality lower than 20 (Q-value≦ 20) were removed. High quality (HQ) clean reads were mapped to ribosome RNA (rRNA) using Bowtie2 by default parameters. The rRNA removed reads were mapped to our previous completed *S. japonica* genome database (NCBI: MEHQ00000000) using TopHat2 v2.0.3.12 software [50]. The parameters are as follows: 1) maximum read mismatch is 2; 2) the distance between mate-pair reads is 50 bp; and 3) the error of distance between mate-pair reads is ±80 bp. The reconstruction of transcripts was performed with Cufflinks with the reference annotation based transcripts (RABT) program [51]. Cuffmerge was used to merge transcripts from different replicas of a group into a set of transcripts, followed by merging the transcripts from multiple groups into a finally comprehensive set of transcripts for downstream differential expression analysis. To identify the new gene transcripts, all of the reconstructed transcripts were aligned to *S. japonica* reference genome and were divided into twelve categories by using Cuffcompare. Genes with class code “u” (the transcripts was unknown or in the intergenic spacer region) were defined as novel genes judged by length longer than 200 bp and exon number > 2.Novel genes were then aligned to the Nr, Swissprot, COG and KEGG databases to obtain protein functional annotation. Raw-reads data were deposited in the NCBI Sequence Read Archive (SRA) with accession number of PRJNA512328.

### Identification of differentially expressed genes (DEGs)

Gene abundances were analyzed with software RSEM [52] and expression levels were normalized by FPKM (Fragments Per Kilobase of transcript per Million mapped reads). EdgeR package (http://www.r-project.org/) was used to identify DEGs across samples [53]. DEGs with fold change > 2 (i.e. ∣log2FC∣ ≥ 1) and false discovery rate (FDR) ≤ 0.05 were considered to be significant. All DEGs were mapped to GO terms in Gene Ontology database (http://www.geneontology.org/). GO terms with corrected *p*-value ≤0.05 were defined as significantly enriched. Kyoto Encyclopedia of Genes and Genomes (KEGG) pathway analysis (*p*-value ≤0.05) enriched metabolic pathways and signal transduction pathways [54]. The heatmap was plotted using the OmicShare tools (http://www.omicshare.com/tools).

### Trend analysis and co-expression network analysis

Gene expression levels in each kelp sample were normalized and clustered by Short Time-series Expression Miner software (STEM) [55]. Clustered profiles with *p*-value ≤0.05 were considered significant. DEGs in each profile were subjected to GO and KEGG enrichment analysis with Q value ≤0.05. The heatmap was plotted using the OmicShare tools. DEGs were used for the weighted gene co-expression network analysis (WGCNA) [56]. Gene expression values were imported into WGCNA (v1.47) using automatic network construction function blockwise Modules with default settings, except that the power is 14. Putative genes were clustered into various correlated modules and those with high connectivity were considered to be hub genes with important functions. Networks were visualized withCytoscape3.3.0software [57]. Significantly enriched GO terms and related pathway in each module were defined with an FDR of < 0.05.

### Detection of alginate and mannitol concentrations

Alginate content was determined according to the method presented in [58]. We immersed 100 mg of ground dry kelp powder in 10 mL 0.4 M H_2_SO_4_ solution overnight, and the mixture was filtered and washed three times with ddH_2_O. Residue and filter paper were transferred into 20 mL 3% Na_2_CO_3_ and incubated at 50 °C water bath overnight. After filtration, the solution was brought to 100 mL by adding ddH_2_O. Subsequently, 1 mL solution was pipetted into a glass cuvette on ice and then 6 mL H_2_SO_4_ was added slowly. The cuvette was immersed in boiling water for 20 min and 3.5 mL from the cuvette was used as negative control group. We added 0.3 mL 0.2% carbazole-ethanol solution to the other 3.5 mL mixture and incubated it at room temperature for 45 min. Absorbance of each 200 μL mixture was detected on the spectrophotometer at 530 nm (Biotek, Winooski, USA). Alginate content was calculated according to the standard curve produced by purchased alginate (Sigma, USA).

Mannitol concentration was measured according to an acetylacetone photocolorimetric method as described in Zeng (2008) [59]. We added 2.5 g of ground dry kelp powder to 80 mL ddH_2_O and boiled in a reflux condenser for 2 h. The filtered solution was brought to 100 mL by washing algal residue with ddH_2_O. Subsequently, 1 mL solution was pipetted to 1 mL 0.015 M NaIO_4_, and incubated at room temperature for 10 min. Two milliliter 0.1% L-rhamnose and 4 mL Nash reagent were added to the mixture and incubated at 53°C for 15 min. Mannitol content was detected at an absorbance of 412 nm (Biotek, Winooski, USA).

### Real-time quantitative PCR (RT-qPCR) verification

Extraction of total RNA and synthesis of the first strand cDNA were performed as described in Zhang et al. (2018) [16]. Five annotated genes (*MPI2*, *PMM1*, *GMD3*, *MC5E70* and *MC5E122*) for alginate biosynthesis and four genes (*M1PDH1*, *M1Pase*, *M2DH* and *FK*) for mannitol metabolism were selected for RT-qPCR verification. β-actin was used as internal control and the PCR procedure was as described in Shao et al. (2014) [25]. Primers used for RT-qPCR are listed in Additional file 16: Table S10.

## Supplementary information

**Additional file 1:** **Figure S1.** Diagrammatic sketch of 4 clipped samples from each individual kelp.

**Additional file 2:** **Table S1.** Statistics on the quality and output of the RNA-Seq data.

**Additional file 3:** **Figure S2**. Ontology enrichment analysis of novel genes.

**Additional file 4:** **Figure S3**. Overview of expression profiles of genes with developmental stages (a) and along the frond (b).

**Additional file 5:** **Table S2**. Expression profiles and their major enriched pathways showing alginate and mannitol contents with development stages and along the frond.

**Additional file 6:** **Table S3**. “*Imm* upregulated 3” genes highly enriched in young and basal blades.

**Additional file 7:** **Figure S4**. Contents of mannitol and alginate in *S. japonica* detected in different developmental stages and tissues from January to June.

**Additional file 8:** **Table S4**. Genes involved in the metabolism of alginate and mannitol in *S. japonica*.

**Additional file 9:** **Figure S5**. Hierarchical cluster tree showing 22 modules of co-expressed genes.

**Additional file 10:** **Table S5**. The statistics of genes in each module.

**Additional file 11:** **Figure S6**. Gene expression pattern in modules of “darkorange” (a), “mediumpurple” (b) and “greenyellow” (c).

**Additional file 12:** **Table S6**. The enriched pathways in “brown4”, “darkgreen”, “black” and “darkslateblue” modules (*p* < 0.05).

**Additional file 13:** **Table S7**. The description of genes in enriched photosynthesis-relevant pathways which are highly correlated with greenyellow module (*p* < 0.05).

**Additional file 14:** **Table S8**. The transcription factors annotated in all the 22 modules.

**Additional file 15:** **Table S9**. The transcription factors correlated with mannitol and alginate biosynthesis.

**Additional file 16:** **Table S10**. Primers of alginate and mannitol relevant genes used for RT-qPCR verification.

## Data Availability

The reference genome of *S. japonica* could be retrieved in GenBank at the National Centre for Biotechnology Information (NCBI) with accession number of MEHQ0000000.1. Raw-reads data were deposited in the NCBI Sequence Read Archive (SRA) with accession number of PRJNA512328.

## Abbreviations

ATPC: ATP synthase gamma chain
Cdc21: Cdc21-like protein
CRYD: Cryptochrome 3
CS: Citrate synthase
CTPS: CTP synthase
DEG: Differentially expressed gene
DNApol: DNA polymerase
E2F: Transcription factor E2F
F6P: Fructose-6-phosphate
FBP: Fructose-1,6-bisphosphatase
FK: Fructokinase
GK: Glucokinase
GMD: GDP-mannose 6-dehydrogenase
GT2: Beta-1,3-glucan synthases (family GT2)
HSF: Heat Shock transcription factor
IL: Isocitrate lyase
KEGG: Kyoto Encyclopedia of Genes and Genomes
LCYB: Lycopene beta cyclase, chloroplast precursor
LHCf6: Light harvesting protein lhcf6
LUT5: Cytochrome P450
M1Pase: Mannitol-1-phosphatase
M1PDH: Mannitol-1-phosphate dehydrogenase
M2DH: Mannitol-2-dehydrogenase
MC5E: Mannuronate C5-epimerase
MDH: Malate dehydrogenase
MPI: Mannose-6-phosphate isomerase
MS: Malate synthase
MYB3R: Myb3R transcription factor
NAD10: NADH dehydrogenase (ubiquinone) subunit 10
NF-Y: Histone-like transcription factor
ODH: Oxoglutarate dehydrogense
PEPCK: Phosphoenolpyruvate carboxykinase
PFK: Pyrophosphate-dependent phosphofructose kinase
PGK: Phosphoglycerate kinase
PHYB: Phytochrome-like protein 3
PK: Pyruvate kinase
PMM: Phosphomannomutase
PsbU: Photosystem II 12 kDa extrinsic protein
RIN: RNA integrity number
RNApol: RNA polymerase II
RP: Ribosomal protein
RT-qPCR: Real-time quantitative PCR
TCA: Tricarboxylic acid
TF: Transcription factor
TFII: Transcription factor II H
Ubi1: Ubiquitin-conjugating enzyme 1
Ubi2: Ubiquitin-conjugating e2 j1
WGCNA: Weighted gene co-expression network analysis
ZDS: Zeta-carotene desaturase, chloroplast precursor
