# Supplementary figures and images for: Transcriptome sequencing of Saccharina japonica sporophytes during whole developmental periods reveals regulatory networks underlying alginate and mannitol biosynthesis
Source: BMC Genomics. 2019 Dec 12;20:975. doi: 10.1186/s12864-019-6366-x (PMC6909449; doi:10.1186/s12864-019-6366-x)

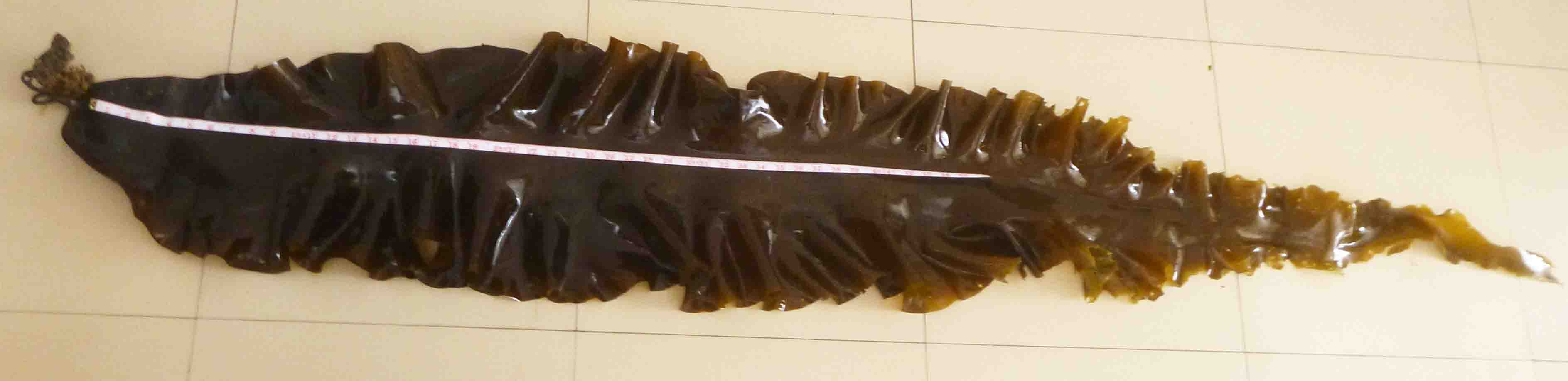


Basal blade

1/3 blade

2/3 blade

distal blade

Fig. S1

Supplement: Supplementary file 1 — Additional file 1: Figure S1. Diagrammatic sketch of 4 clipped samples from each individual kelp. [file 12864_2019_6366_MOESM1_ESM.docx]

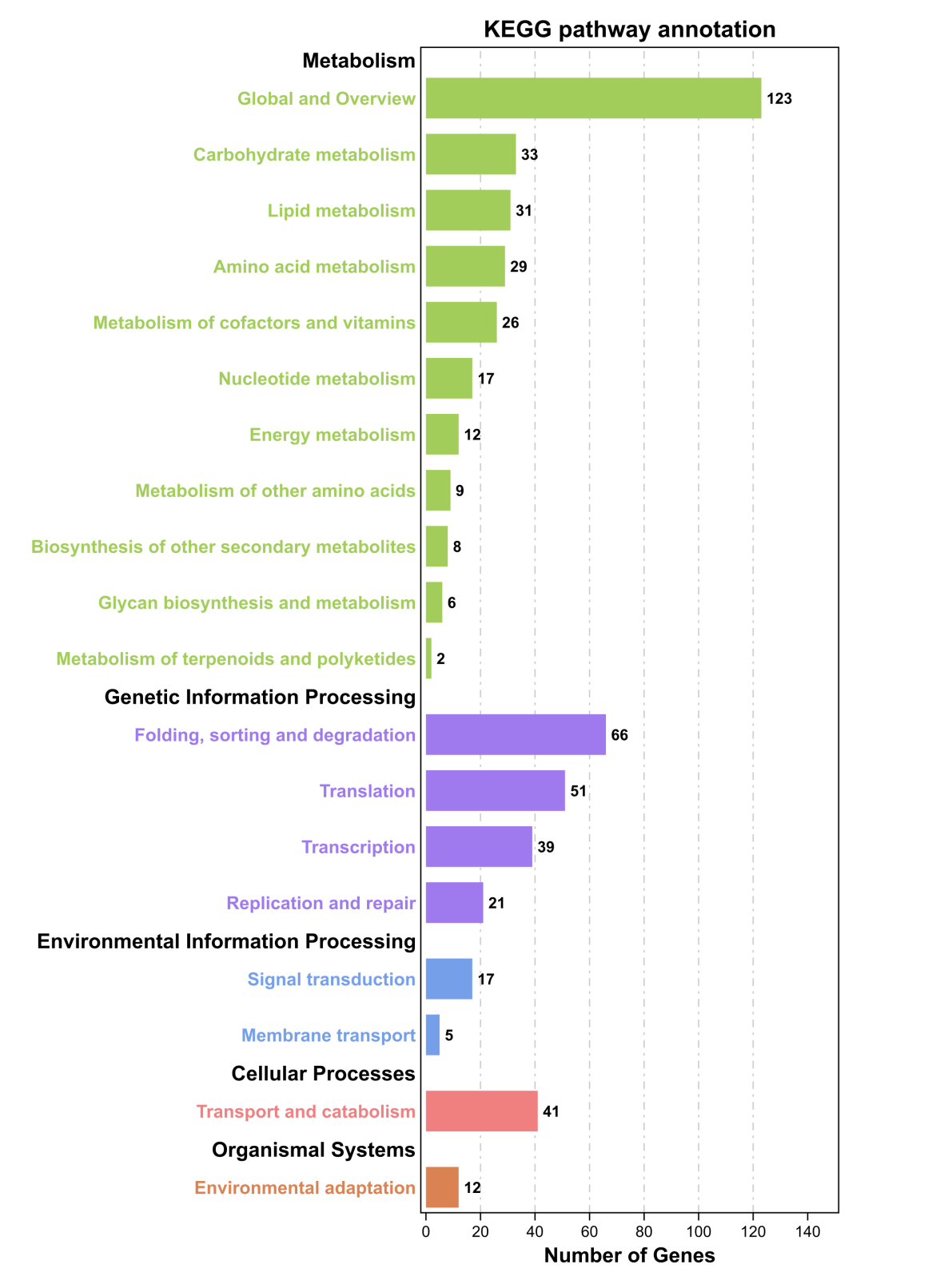


Fig. S2

Supplement: Supplementary file 3 — Additional file 3: Figure S2. Ontology enrichment analysis of novel genes. [file 12864_2019_6366_MOESM3_ESM.docx]

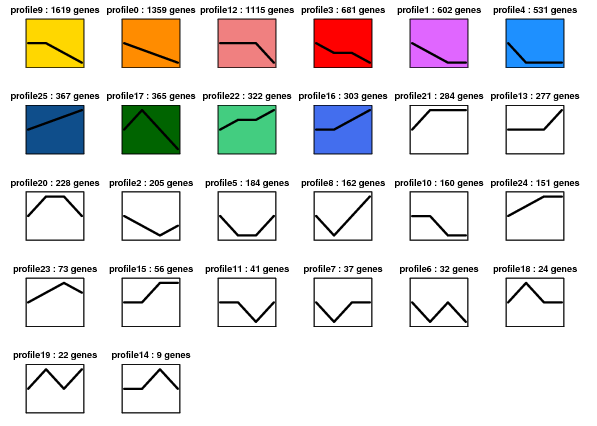

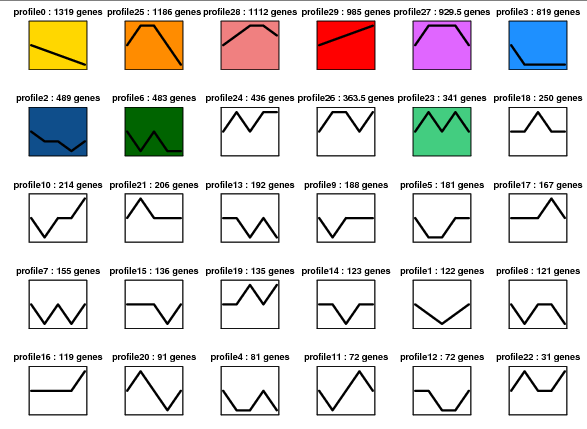


b

a

Fig. S3

Supplement: Supplementary file 4 — Additional file 4: Figure S3. Overview of expression profiles of genes with developmental stages (a) and along the frond (b). [file 12864_2019_6366_MOESM4_ESM.docx]

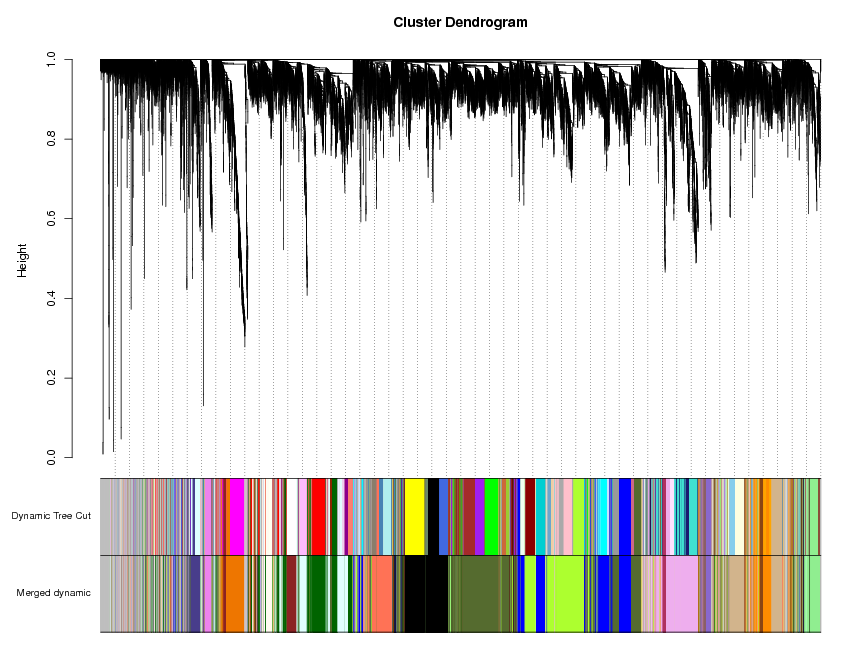


Fig. S5

Supplement: Supplementary file 9 — Additional file 9: Figure S5. Hierarchical cluster tree showing 22 modules of co-expressed genes. [file 12864_2019_6366_MOESM9_ESM.docx]

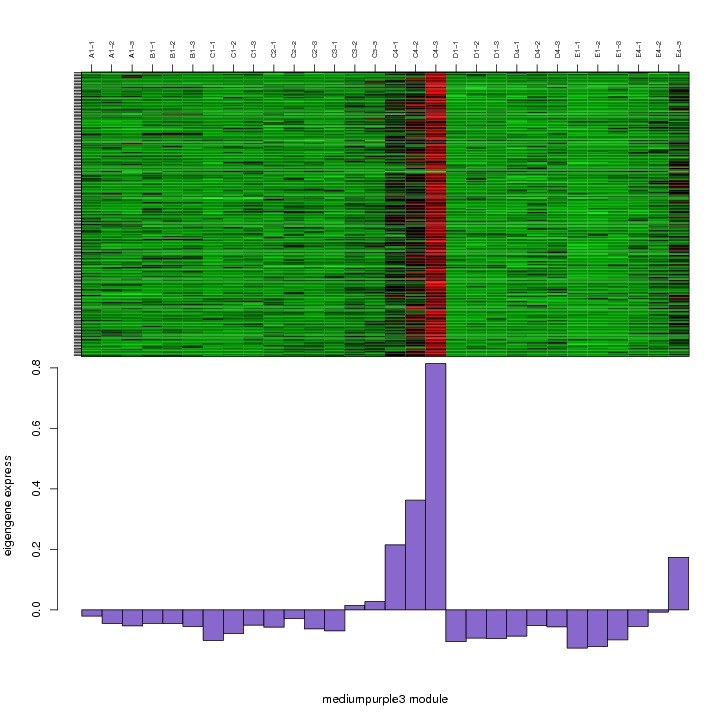

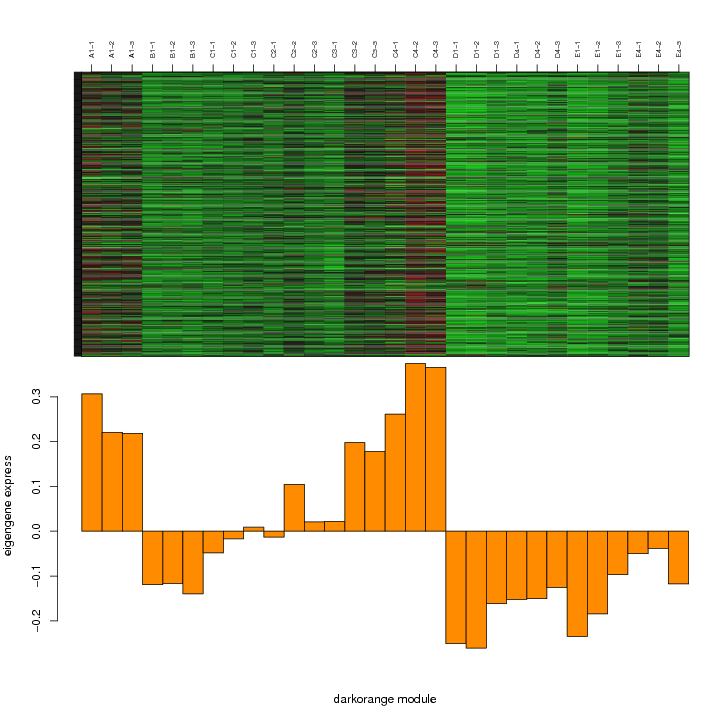


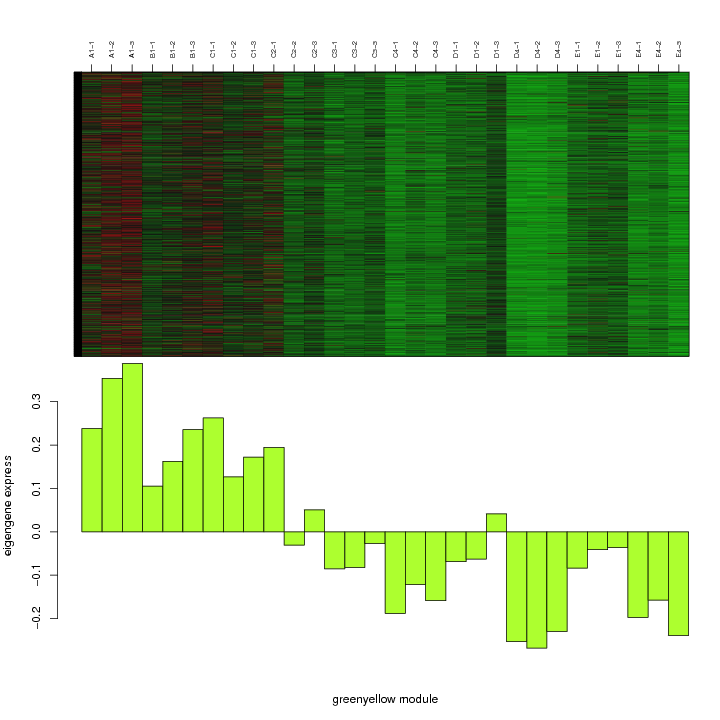


**Greenyellow**

**Darkorange**

**Mediumpurple**

a

b

c

Fig. S6

Supplement: Supplementary file 11 — Additional file 11: Figure S6. Gene expression pattern in modules of “darkorange” (a), “mediumpurple” (b) and “greenyellow” (c). [file 12864_2019_6366_MOESM11_ESM.docx]
