# Supplementary material for: Transcriptome sequencing of Saccharina japonica sporophytes during whole developmental periods reveals regulatory networks underlying alginate and mannitol biosynthesis
Source: BMC Genomics. 2019 Dec 12;20:975. doi: 10.1186/s12864-019-6366-x (PMC6909449; doi:10.1186/s12864-019-6366-x)
Supplement: Supplementary file 2 — Additional file 2: Table S1. Statistics on the quality and output of the RNA-Seq data. [file 12864_2019_6366_MOESM2_ESM.docx]

| Table S1 Statistics on the quality and output of the RNA-Seq data | | | | | | | | | | | | | | | |
| --- | --- | --- | --- | --- | --- | --- | --- | --- | --- | --- | --- | --- | --- | --- | --- |
| Sample | RIN | Clean Data(bp) | HQ Clean Data(bp) | Q30(%) | N(%) | GC(%) | Clean reads num | HQ clean reads num (%) | Total reads unmapped rRNA num | Unique mapped reads | multiple mapped reads | Mapping Ratio | Known Gene Num | New Gene Num | All Gene Num |
| JaB1-1 | 8.2 | 8422201500 | 8105889861 | 7704876714 (95.05%) | 101600 (0.00%) | 4521560023 (55.78%) | 56148010 | 55129120 (98.19%) | 54559876 | 42983061 (78.78%) | 1383734 (2.54%) | 81.32% | 16160 (66.18%) | 3695 | 19855 |
| JaB1-2 | 7.9 | 8277855000 | 7955514409 | 7573414947 (95.20%) | 102944 (0.00%) | 4437556009 (55.78%) | 55185700 | 54136460 (98.1%) | 53927050 | 42014626 (77.91%) | 1532272 (2.84%) | 80.75% | 15747 (64.49%) | 3514 | 19261 |
| JaB1-3 | 8.3 | 7732006800 | 7443626257 | 7096044641 (95.33%) | 94398 (0.00%) | 4220091622 (56.69%) | 51546712 | 50598558 (98.16%) | 50436890 | 40055759 (79.42%) | 1316418 (2.61%) | 82.03% | 15976 (65.42%) | 3609 | 19585 |
| MhB-1 | 8.5 | 8659647300 | 8364554679 | 8038038072 (96.10%) | 141594 (0.00%) | 4757476362 (56.88%) | 57730982 | 56753366 (98.31%) | 55396890 | 43965713 (79.36%) | 1307870 (2.36%) | 81.73% | 16543 (67.75%) | 3838 | 20381 |
| MhB-2 | 8.8 | 8905520100 | 8590638397 | 8247396117 (96.00%) | 146420 (0.00%) | 4886491651 (56.88%) | 59370134 | 58311338 (98.22%) | 55132620 | 43667049 (79.20%) | 1406672 (2.55%) | 81.76% | 16791 (68.76%) | 3863 | 20654 |
| MhB-3 | 8.7 | 12724976400 | 12214001205 | 11654882354 (95.42%) | 202494 (0.00%) | 6948107338 (56.89%) | 84833176 | 82982428 (97.82%) | 80513848 | 63178597 (78.47%) | 1839342 (2.28%) | 80.75% | 17120 (70.11%) | 3900 | 21020 |
| ApB-1 | 8.3 | 9168525000 | 8836979560 | 8474158951 (95.89%) | 150595 (0.00%) | 5031818556 (56.94%) | 61123500 | 59995464 (98.15%) | 58760544 | 46469793 (79.08%) | 1294110 (2.20%) | 81.29% | 16443 (67.34%) | 3771 | 20214 |
| ApB-2 | 7.6 | 6852831900 | 6606166213 | 6342555818 (96.01%) | 103803 (0.00%) | 3681116267 (55.72%) | 45685546 | 44857256 (98.19%) | 40670556 | 32073052 (78.86%) | 808714 (1.99%) | 80.85% | 16028 (65.64%) | 3657 | 19685 |
| ApB-3 | 7.2 | 9427189800 | 9083236696 | 8714986821 (95.95%) | 147169 (0.00%) | 5098499979 (56.13%) | 62847932 | 61712556 (98.19%) | 61419158 | 48543896 (79.04%) | 1225186 (1.99%) | 81.03% | 16767 (68.66%) | 3834 | 20601 |
| Ap1-1 | 7.8 | 8766293100 | 8451142682 | 8109707286 (95.96%) | 132657 (0.00%) | 4789554877 (56.67%) | 58441954 | 57375158 (98.17%) | 57011316 | 45021150 (78.97%) | 1246056 (2.19%) | 81.15% | 16340 (66.92%) | 3708 | 20048 |
| Ap1-2 | 7.5 | 8415159600 | 8112048085 | 7780748584 (95.92%) | 134806 (0.00%) | 4566572198 (56.29%) | 56101064 | 55094228 (98.21%) | 53500450 | 42754146 (79.91%) | 1263282 (2.36%) | 82.27% | 15807 (64.73%) | 3577 | 19384 |
| Ap1-3 | 7.4 | 12549726600 | 11959266673 | 11546449101 (96.55%) | 202672 (0.00%) | 6790793610 (56.78%) | 83664844 | 82250510 (98.31%) | 77255732 | 38002174 (49.19%) | 982926 (1.27%) | 50.46% | 15906 (65.14%) | 3625 | 19531 |
| Ap2-1 | 7.6 | 9345864600 | 9023312450 | 8710094102 (96.53%) | 165872 (0.00%) | 5051744049 (55.99%) | 62305764 | 61201300 (98.23%) | 60600628 | 47891292 (79.03%) | 1340138 (2.21%) | 81.24% | 15958 (65.35%) | 3566 | 19524 |
| Ap2-2 | 7.6 | 8328839700 | 8085648936 | 7813546505 (96.63%) | 148020 (0.00%) | 4579216689 (56.63%) | 55525598 | 54676666 (98.47%) | 54276166 | 43553015 (80.24%) | 1225654 (2.26%) | 82.50% | 15520 (63.56%) | 3524 | 19044 |
| Ap2-3 | 7.9 | 10116588600 | 9748566690 | 9389238517 (96.31%) | 179563 (0.00%) | 5471863612 (56.13%) | 67443924 | 66137200 (98.06%) | 65721522 | 51942372 (79.03%) | 1543724 (2.35%) | 81.38% | 16292 (66.72%) | 3652 | 19944 |
| ApD-1 | 7.9 | 9285897600 | 8977772176 | 8671788973 (96.59%) | 164474 (0.00%) | 5012846201 (55.84%) | 61905984 | 60844024 (98.28%) | 59936484 | 47359632 (79.02%) | 1450662 (2.42%) | 81.44% | 15472 (63.36%) | 3467 | 18939 |
| ApD-2 | 7.6 | 8717790900 | 8431515143 | 8134478553 (96.48%) | 155901 (0.00%) | 4762008465 (56.48%) | 58118606 | 57116412 (98.28%) | 56602740 | 45137127 (79.74%) | 1242354 (2.19%) | 81.94% | 15288 (62.61%) | 3462 | 18750 |
| ApD-3 | 8 | 9020648700 | 8725284277 | 8432252898 (96.64%) | 159760 (0.00%) | 4901475391 (56.18%) | 60137658 | 59127420 (98.32%) | 58381374 | 45656321 (78.20%) | 1293304 (2.22%) | 80.42% | 15584 (63.82%) | 3452 | 19036 |
| MyB-1 | 7.9 | 8463852000 | 8192717463 | 7921696371 (96.69%) | 149775 (0.00%) | 4597774388 (56.12%) | 56425680 | 55511462 (98.38%) | 52356216 | 40815422 (77.96%) | 1285098 (2.45%) | 80.41% | 16210 (66.38%) | 3704 | 19914 |
| MyB-2 | 8.1 | 8122281000 | 7859837839 | 7589704362 (96.56%) | 145135 (0.00%) | 4439871640 (56.49%) | 54148540 | 53247966 (98.34%) | 52021746 | 40927398 (78.67%) | 1108824 (2.13%) | 80.81% | 16622 (68.07%) | 3779 | 20401 |
| MyB-3 | 8.1 | 8797548300 | 8501378136 | 8200714136 (96.46%) | 156085 (0.00%) | 4798390943 (56.44%) | 58650322 | 57605504 (98.22%) | 54766972 | 43040945 (78.59%) | 1284938 (2.35%) | 80.94% | 16367 (67.03%) | 3778 | 20145 |
| MyD-1 | 8.1 | 7689583800 | 7447500844 | 7195682800 (96.62%) | 135241 (0.00%) | 4250481240 (57.07%) | 51263892 | 50411628 (98.34%) | 49033516 | 39141226 (79.83%) | 1000570 (2.04%) | 81.87% | 15346 (62.84%) | 3367 | 18713 |
| MyD-2 | 8 | 7842135000 | 7583078316 | 7321934952 (96.56%) | 138033 (0.00%) | 4311142078 (56.85%) | 52280900 | 51360032 (98.24%) | 50783734 | 40450113 (79.65%) | 990312 (1.95%) | 81.60% | 15098 (61.83%) | 3359 | 18457 |
| MyD-3 | 7.3 | 8761026300 | 8482790919 | 8200780308 (96.68%) | 154761 (0.00%) | 4813210312 (56.74%) | 58406842 | 57460676 (98.38%) | 54337852 | 43074021 (79.27%) | 1050518 (1.93%) | 81.20% | 15016 (61.49%) | 3357 | 18373 |
| JuB-1 | 8.2 | 8991723900 | 8620800356 | 8196765858 (95.08%) | 110042 (0.00%) | 4936027423 (57.26%) | 59944826 | 58701872 (97.93%) | 58158964 | 46855801 (80.57%) | 1275932 (2.19%) | 82.76% | 15985 (65.46%) | 3642 | 19627 |
| JuB-2 | 8 | 8991994200 | 8664161104 | 8270093286 (95.45%) | 111403 (0.00%) | 4913031720 (56.71%) | 59946628 | 58872802 (98.21%) | 56097220 | 44743202 (79.76%) | 1213580 (2.16%) | 81.92% | 16124 (66.03%) | 3644 | 19768 |
| JuB-3 | 9.2 | 9361543500 | 9015927323 | 8599673460 (95.38%) | 116306 (0.00%) | 5131557351 (56.92%) | 62410290 | 61286010 (98.2%) | 60175942 | 48121049 (79.97%) | 1386882 (2.30%) | 82.27% | 15718 (64.37%) | 3566 | 19284 |
| JuD-1 | 8 | 10215351600 | 9828671187 | 9366223536 (95.29%) | 127202 (0.00%) | 5614630131 (57.13%) | 68102344 | 66808478 (98.1%) | 66209192 | 53007191 (80.06%) | 1143818 (1.73%) | 81.79% | 15883 (65.04%) | 3531 | 19414 |
| JuD-2 | 7.7 | 8313680400 | 7992683039 | 7612734074 (95.25%) | 102536 (0.00%) | 4572544054 (57.21%) | 55424536 | 54324726 (98.02%) | 54069556 | 42941219 (79.42%) | 1114794 (2.06%) | 81.48% | 15707 (64.32%) | 3575 | 19282 |
| JuD-3 | 8 | 7239394800 | 6971438487 | 6651379974 (95.41%) | 90607 (0.00%) | 3909386058 (56.08%) | 48262632 | 47371766 (98.15%) | 47047714 | 36050395 (76.63%) | 916434 (1.95%) | 78.57% | 15028 (61.54%) | 3375 | 18403 |
