# Supplementary material for: Transcriptome sequencing of Saccharina japonica sporophytes during whole developmental periods reveals regulatory networks underlying alginate and mannitol biosynthesis
Source: BMC Genomics. 2019 Dec 12;20:975. doi: 10.1186/s12864-019-6366-x (PMC6909449; doi:10.1186/s12864-019-6366-x)
Supplement: Supplementary file 5 — Additional file 5: Table S2. Expression profiles and their major enriched pathways showing alginate and mannitol contents with development stages and along the frond. [file 12864_2019_6366_MOESM5_ESM.docx]

| Table S2 Expression profiles and their major enriched pathways showing alginate and mannitol contents with development stages and along the frond. | | | | | | | | |  |
| --- | --- | --- | --- | --- | --- | --- | --- | --- | --- |
| From Jan to Jun | Pathway (Genes numbers) | Pvalue | Qvalue |  | From base to tip | Pathway (Genes numbers) | Pvalue | Qvalue |  |
| profile29: 985 genes | Ribosome (20) | 0 | 0 |  | profile25: 367 genes | ABC transporters (4) | 0.0047 | 0.1519 |  |
| 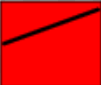   \|  \| \| --- \| | Homologous recombination (4) | 0.030462 | 0.583466 |  | 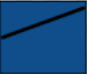 | RNA transport (6) | 0.0074 | 0.1519 |  |
|  | Nitrogen metabolism (3) | 0.042298 | 0.583466 |  |  | Photosynthesis (2) | 0.03 | 0.4094 |  |
|  |  |  |  |  |  | Amino sugar and nucleotide sugar metabolism (3) | 0.0437 | 0.4476 |  |
|  |  |  |  |  |  |  |  |  |  |
|  |  |  |  |  |  |  |  |  |  |
|  |  |  |  |  |  |  |  |  |  |
| profile0: 1319 genes | Oxidative phosphorylation (18) | 0 | 0.000006 |  | profile22: 322 genes | [Arachidonic acid metabolism (4)](file:///D:\Programs\ProductionJournal\Temp\Table%20S1-S10%2020191105%20BMC.xlsx#RANGE!gene1) | 0.002 | 0.0877 |  |
| 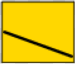   \|  \| \| --- \| | [Photosynthesis - antenna proteins (12)](file:///D:\Programs\ProductionJournal\Temp\Table%20S1-S10%2020191105%20BMC.xlsx#RANGE!gene2) | 0.000024 | 0.000752 |  | 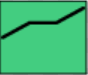 | [Citrate cycle (TCA cycle) (4)](file:///D:\Programs\ProductionJournal\Temp\Table%20S1-S10%2020191105%20BMC.xlsx#RANGE!gene2) | 0.0038 | 0.0877 |  |
|  | [Phagosome (13)](file:///D:\Programs\ProductionJournal\Temp\Table%20S1-S10%2020191105%20BMC.xlsx#RANGE!gene3) | 0.000025 | 0.000752 |  |  | [Glutathione metabolism (5)](file:///D:\Programs\ProductionJournal\Temp\Table%20S1-S10%2020191105%20BMC.xlsx#RANGE!gene3) | 0.0048 | 0.0877 |  |
|  | [Proteasome (14)](file:///D:\Programs\ProductionJournal\Temp\Table%20S1-S10%2020191105%20BMC.xlsx#RANGE!gene4) | 0.000032 | 0.000752 |  |  | [Diterpenoid biosynthesis (1)](file:///D:\Programs\ProductionJournal\Temp\Table%20S1-S10%2020191105%20BMC.xlsx#RANGE!gene4) | 0.0174 | 0.1981 |  |
|  | Photosynthesis (7) | 0.00024 | 0.004461 |  |  | [Pyruvate metabolism (4)](file:///D:\Programs\ProductionJournal\Temp\Table%20S1-S10%2020191105%20BMC.xlsx#RANGE!gene5) | 0.018 | 0.1981 |  |
|  | [Carbon fixation in photosynthetic organisms (11)](file:///D:\Programs\ProductionJournal\Temp\Table%20S1-S10%2020191105%20BMC.xlsx#RANGE!gene6) | 0.00124 | 0.019218 |  |  | [beta-Alanine metabolism (2)](file:///D:\Programs\ProductionJournal\Temp\Table%20S1-S10%2020191105%20BMC.xlsx#RANGE!gene6) | 0.0378 | 0.3167 |  |
|  | [Metabolic pathways (106)](file:///D:\Programs\ProductionJournal\Temp\Table%20S1-S10%2020191105%20BMC.xlsx#RANGE!gene7) | 0.003636 | 0.048307 |  |  | [Microbial metabolism in diverse environments (8)](file:///D:\Programs\ProductionJournal\Temp\Table%20S1-S10%2020191105%20BMC.xlsx#RANGE!gene7) | 0.0403 | 0.3167 |  |
|  | [Microbial metabolism in diverse environments (30)](file:///D:\Programs\ProductionJournal\Temp\Table%20S1-S10%2020191105%20BMC.xlsx#RANGE!gene8) | 0.006742 | 0.078381 |  | profile16: 303 genes |  |  |  |  |
|  | [Fructose and mannose metabolism (7)](file:///D:\Programs\ProductionJournal\Temp\Table%20S1-S10%2020191105%20BMC.xlsx#RANGE!gene9) | 0.019504 | 0.185083 |  |  | Linoleic acid metabolism (3) | 0.0003 | 0.015 |  |
|  | [Biosynthesis of antibiotics (35)](file:///D:\Programs\ProductionJournal\Temp\Table%20S1-S10%2020191105%20BMC.xlsx#RANGE!gene10) | 0.019901 | 0.185083 |  | 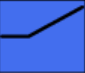 | [Arachidonic acid metabolism (4)](file:///D:\Programs\ProductionJournal\Temp\Table%20S1-S10%2020191105%20BMC.xlsx#RANGE!gene2) | 0.002 | 0.058 |  |
|  | [Biosynthesis of secondary metabolites (55)](file:///D:\Programs\ProductionJournal\Temp\Table%20S1-S10%2020191105%20BMC.xlsx#RANGE!gene11) | 0.033749 | 0.285335 |  |  | [Cysteine and methionine metabolism (4)](file:///D:\Programs\ProductionJournal\Temp\Table%20S1-S10%2020191105%20BMC.xlsx#RANGE!gene3) | 0.0055 | 0.1061 |  |
|  |  |  |  |  |  | [Biosynthesis of amino acids (7)](file:///D:\Programs\ProductionJournal\Temp\Table%20S1-S10%2020191105%20BMC.xlsx#RANGE!gene4) | 0.0169 | 0.2196 |  |
|  |  |  |  |  |  | [Carbon metabolism (7)](file:///D:\Programs\ProductionJournal\Temp\Table%20S1-S10%2020191105%20BMC.xlsx#RANGE!gene5) | 0.0217 | 0.2196 |  |
|  |  |  |  |  |  | [Metabolic pathways (25)](file:///D:\Programs\ProductionJournal\Temp\Table%20S1-S10%2020191105%20BMC.xlsx#RANGE!gene6) | 0.0258 | 0.2196 |  |
|  |  |  |  |  |  | [Citrate cycle (TCA cycle) (3)](file:///D:\Programs\ProductionJournal\Temp\Table%20S1-S10%2020191105%20BMC.xlsx#RANGE!gene7) | 0.027 | 0.2196 |  |
|  |  |  |  |  |  | [SNARE interactions in vesicular transport (2)](file:///D:\Programs\ProductionJournal\Temp\Table%20S1-S10%2020191105%20BMC.xlsx#RANGE!gene8) | 0.0303 | 0.2196 |  |
|  |  |  |  |  |  | [ABC transporters (3)](file:///D:\Programs\ProductionJournal\Temp\Table%20S1-S10%2020191105%20BMC.xlsx#RANGE!gene9) | 0.0372 | 0.2397 |  |
|  |  |  |  |  |  | [Pentose and glucuronate interconversions (2)](file:///D:\Programs\ProductionJournal\Temp\Table%20S1-S10%2020191105%20BMC.xlsx#RANGE!gene10) | 0.0459 | 0.2663 |  |
|  |  |  |  |  | profile9: 1619 genes |  |  |  |  |
|  |  |  |  |  | 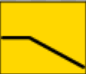 | Photosynthesis - antenna proteins (15) | 0 | 6E-06 |  |
|  |  |  |  |  |  | Metabolic pathways (114) | 3E-05 | 0.0015 |  |
|  |  |  |  |  |  | Microbial metabolism in diverse environments (32) | 0.0014 | 0.04 |  |
|  |  |  |  |  |  | Biosynthesis of secondary metabolites (61) | 0.0017 | 0.04 |  |
|  |  |  |  |  |  | Thiamine metabolism (4) | 0.0024 | 0.0464 |  |
|  |  |  |  |  |  |  |  |  |  |
|  |  |  |  |  |  |  |  |  |  |
|  |  |  |  |  |  | Metabolic pathways (91) | 0.0012 | 0.0538 |  |
|  |  |  |  |  | profile0: 1359 genes | Microbial metabolism in diverse environments (28) | 0.0013 | 0.0538 |  |
|  |  |  |  |  |  | Pyruvate metabolism (11) | 0.0017 | 0.0538 |  |
|  |  |  |  |  | 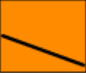 | Valine, leucine and isoleucine degradation (9) | 0.0037 | 0.0876 |  |
|  |  |  |  |  |  | Alanine, aspartate and glutamate metabolism (7) | 0.0057 | 0.1081 |  |
|  |  |  |  |  |  | Carbon fixation in photosynthetic organisms (8) | 0.0127 | 0.1707 |  |
|  |  |  |  |  |  | Citrate cycle (TCA cycle) (7) | 0.0127 | 0.1707 |  |
|  |  |  |  |  |  | Carbon metabolism (19) | 0.0149 | 0.1752 |  |
|  |  |  |  |  |  |  |  |  |  |
