# Supplementary material for: Transcriptome sequencing of Saccharina japonica sporophytes during whole developmental periods reveals regulatory networks underlying alginate and mannitol biosynthesis
Source: BMC Genomics. 2019 Dec 12;20:975. doi: 10.1186/s12864-019-6366-x (PMC6909449; doi:10.1186/s12864-019-6366-x)
Supplement: Supplementary file 6 — Additional file 6: Table S3. “Imm upregulated 3” genes highly enriched in young and basal blades. [file 12864_2019_6366_MOESM6_ESM.docx]

| Table S3 *Imm* upregulated 3 genes highly enriched in young and basal blades. | | | | | | | | | | |
| --- | --- | --- | --- | --- | --- | --- | --- | --- | --- | --- |
| GeneID | SPOT | Profile | JaB | MhB | ApB | MyB | JuB | Symbol | Description |  |
| GENE_008015 | ID_108 | 0 | 0 | 0.88 | -1.89 | -4.56 | -10.1 | - | imm upregulated 3 [Ectocarpus siliculosus] | |
| GENE_008057 | ID_2193 | 0 | 0 | -1.33 | -2.69 | -2.16 | -6.5 | - | imm upregulated 3 [Ectocarpus siliculosus] | |
| GENE_008224 | ID_2296 | 0 | 0 | 0.5 | -2.11 | -3.23 | -5.17 | - | imm upregulated 3 [Ectocarpus siliculosus] | |
| GENE_013917 | ID_2372 | 0 | 0 | -1.46 | -2.42 | -1.62 | -8.15 | - | imm upregulated 3 [Ectocarpus siliculosus] | |
| GENE_017497 | ID_4053 | 0 | 0 | -1.39 | -4.45 | -7.91 | -7.61 | - | imm upregulated 3 [Ectocarpus siliculosus] | |
| GENE_019496 | ID_5176 | 0 | 0 | -1.26 | -4.22 | -4.46 | -5.33 | - | imm upregulated 3 [Ectocarpus siliculosus] | |
| GENE_019741 | ID_5741 | 0 | 0 | 0.06 | -1.11 | -1.01 | -4.5 | - | imm upregulated 3 [Ectocarpus siliculosus] | |
| GENE_019910 | ID_5806 | 0 | 0 | -3.51 | -5.27 | -8.48 | -10.62 | - | imm upregulated 3 [Ectocarpus siliculosus] | |
| GENE_020314 | ID_5850 | 0 | 0 | -1.38 | -1.67 | -3.56 | -4.22 | - | imm upregulated 3 [Ectocarpus siliculosus] | |
| GENE_022151 | ID_5989 | 0 | 0 | -2.04 | -3.03 | -4.52 | -6.57 | - | imm upregulated 3 [Ectocarpus siliculosus] | |
| GENE_022676 | ID_6552 | 0 | 0 | -4.55 | -5 | -4.57 | -7.71 | - | imm upregulated 3 [Ectocarpus siliculosus] | |
| GENE_026422 | ID_6682 | 0 | 0 | 0.55 | -1.79 | -3.77 | -11.12 | - | imm upregulated 3 [Ectocarpus siliculosus] | |
| GENE_026539 | ID_7814 | 0 | 0 | -2.46 | -3.33 | -2.79 | -8.31 | Cpn2 | imm upregulated 3 [Ectocarpus siliculosus] | |
| GENE_026540 | ID_7840 | 0 | 0 | -0.36 | -2.38 | -3 | -7.57 | - | imm upregulated 3 [Ectocarpus siliculosus] | |
| GENE_026749 | ID_7841 | 0 | 0 | -0.75 | -1.85 | -3.98 | -6.42 | - | imm upregulated 3 [Ectocarpus siliculosus] | |
| GENE_026751 | ID_7891 | 0 | 0 | -2.25 | -6.57 | -6.57 | -9.31 | - | imm upregulated 3 [Ectocarpus siliculosus] | |
| GENE_026849 | ID_7892 | 0 | 0 | -1.37 | -4.36 | -8.52 | -9.52 | - | imm upregulated 3 [Ectocarpus siliculosus] | |
| GENE_027694 | ID_7918 | 0 | 0 | -3.56 | -5.83 | -9.12 | -12.85 | - | imm upregulated 3 [Ectocarpus siliculosus] | |
| GENE_027995 | ID_8196 | 0 | 0 | 0.47 | -1.22 | -2.42 | -9.86 | - | imm upregulated 3 [Ectocarpus siliculosus] | |
| GENE_028319 | ID_8292 | 0 | 0 | -2.87 | -2.35 | -2.5 | -7.41 | - | imm upregulated 3 [Ectocarpus siliculosus] | |
| GENE_028535 | ID_8386 | 0 | 0 | -1.48 | -2.61 | -5.7 | -6.03 | - | imm upregulated 3 [Ectocarpus siliculosus] | |
| GENE_029159 | ID_8438 | 0 | 0 | -1.46 | -2.07 | -5.41 | -8.87 | - | imm upregulated 3 [Ectocarpus siliculosus] | |
| XLOC_001271 | ID_8614 | 0 | 0 | -2 | -2.79 | -2.25 | -7.98 | - | imm upregulated 3 [Ectocarpus siliculosus] | |
| XLOC_004223 | ID_8900 | 0 | 0 | -5.72 | -6.21 | -4.95 | -10.27 | - | imm upregulated 3 [Ectocarpus siliculosus] | |
| XLOC_013661 | ID_9066 | 0 | 0 | -0.44 | -0.85 | -1.98 | -5.6 | - | imm upregulated 3 [Ectocarpus siliculosus] | |
| XLOC_019543 | ID_9580 | 0 | 0 | -0.85 | -2.14 | -1.72 | -3.28 | - | imm upregulated 3 [Ectocarpus siliculosus] | |
| XLOC_030633 | ID_9914 | 0 | 0 | -2.52 | -7.85 | -6.91 | -7.35 | - | imm upregulated 3 [Ectocarpus siliculosus] | |
| GENE_004400 | ID_10626 | 0 | 0 | -2.98 | -2.52 | -5.03 | -5.84 | - | imm upregulated 3 [Ectocarpus siliculosus] | |
|  |  |  |  |  |  |  |  |  |  |  |
| GeneID | SPOT | Profile | ApB | Ap1 | Ap2 | ApD | Symbol | Description |  |  |
| GENE_017186 | ID_4215 | 0 | 0 | -1.1 | -3.22 | -4.46 | CPN2 | imm upregulated 3 [Ectocarpus siliculosus] | | |
| GENE_018611 | ID_4574 | 0 | 0 | -0.95 | -1.89 | -3.31 | - | imm upregulated 3 [Ectocarpus siliculosus] | | |
| GENE_019496 | ID_4774 | 0 | 0 | -4.93 | -6.73 | -11.05 | - | imm upregulated 3 [Ectocarpus siliculosus] | | |
| GENE_020323 | ID_4962 | 0 | 0 | -0.55 | -1.16 | -1.67 | - | imm upregulated 3 [Ectocarpus siliculosus] | | |
| GENE_020325 | ID_4963 | 0 | 0 | -0.36 | -1.35 | -2.07 | - | imm upregulated 3 [Ectocarpus siliculosus] | | |
| GENE_021452 | ID_5233 | 0 | 0 | -2.31 | -5.49 | -7.9 | - | imm upregulated 3 [Ectocarpus siliculosus] | | |
| XLOC_015960 | ID_8090 | 0 | 0 | -2.42 | -7.36 | -10.68 | - | imm upregulated 3 [Ectocarpus siliculosus] | | |
| GENE_000733 | ID_161 | 1 | 0 | -7.77 | -11.5 | -11.5 | - | imm upregulated 3 [Ectocarpus siliculosus] | | |
| GENE_016547 | ID_4054 | 1 | 0 | -6.7 | -11.6 | -11.6 | - | imm upregulated 3 [Ectocarpus siliculosus] | | |
| GENE_019910 | ID_4859 | 1 | 0 | -3.6 | -4.9 | -5.27 | - | imm upregulated 3 [Ectocarpus siliculosus] | | |
| GENE_026849 | ID_6644 | 1 | 0 | -3.7 | -7.02 | -7.02 | - | imm upregulated 3 [Ectocarpus siliculosus] | | |
| GENE_027133 | ID_6716 | 1 | 0 | -5.52 | -10.72 | -10.72 | - | imm upregulated 3 [Ectocarpus siliculosus] | | |
| XLOC_004223 | ID_7546 | 1 | 0 | -3.09 | -9.08 | -9.08 | - | imm upregulated 3 [Ectocarpus siliculosus] | | |
| XLOC_004635 | ID_7561 | 1 | 0 | -5.07 | -7.8 | -7.8 | - | imm upregulated 3 [Ectocarpus siliculosus] | | |
| XLOC_025922 | ID_8548 | 1 | 0 | -5.92 | -11.83 | -11.83 | LGR4 | imm upregulated 3 [Ectocarpus siliculosus] | | |
| GENE_009201 | ID_2233 | 2 | 0 | -4.84 | -10.04 | -6.3 | - | imm upregulated 3 [Ectocarpus siliculosus] | | |
| GENE_017819 | ID_4375 | 2 | 0 | -3.2 | -6.96 | -2.45 | - | imm upregulated 3 [Ectocarpus siliculosus] | | |
| GENE_022685 | ID_5574 | 2 | 0 | -4.6 | -11.55 | -5.17 | - | imm upregulated 3 [Ectocarpus siliculosus] | | |
| XLOC_017531 | ID_8167 | 2 | 0 | -4.91 | -9.23 | -6.49 | - | imm upregulated 3 [Ectocarpus siliculosus] | | |
| GENE_003538 | ID_805 | 3 | 0 | -5.86 | -6.94 | -9.5 | SLIT2 | imm upregulated 3 [Ectocarpus siliculosus] | | |
| GENE_010695 | ID_2602 | 3 | 0 | -7.24 | -8.98 | -12.3 | - | imm upregulated 3 [Ectocarpus siliculosus] | | |
| GENE_020314 | ID_4960 | 3 | 0 | -0.84 | -0.73 | -1.68 | - | imm upregulated 3 [Ectocarpus siliculosus] | | |
| GENE_022151 | ID_5454 | 3 | 0 | -3.71 | -4.02 | -5.5 | - | imm upregulated 3 [Ectocarpus siliculosus] | | |
| GENE_028319 | ID_6979 | 3 | 0 | -2.35 | -1.76 | -3.8 | - | imm upregulated 3 [Ectocarpus siliculosus] | | |
| GENE_028535 | ID_7028 | 3 | 0 | -2.02 | -2.7 | -6.8 | - | imm upregulated 3 [Ectocarpus siliculosus] | | |
| XLOC_029372 | ID_8745 | 3 | 0 | -3.38 | -3.38 | -6.04 | - | imm upregulated 3 [Ectocarpus siliculosus] | | |
| GENE_009561 | ID_2309 | 4 | 0 | -2.85 | -2.89 | -3.29 | - | imm upregulated 3 [Ectocarpus siliculosus] | | |
| GENE_013917 | ID_3383 | 4 | 0 | -3.74 | -4.64 | -5.04 | - | imm upregulated 3 [Ectocarpus siliculosus] | | |
| GENE_022676 | ID_5572 | 4 | 0 | -3.5 | -4.52 | -4.82 | - | imm upregulated 3 [Ectocarpus siliculosus] | | |
| GENE_020329 | ID_4964 | 12 | 0 | 0.24 | -0.06 | -1.53 | - | imm upregulated 3 [Ectocarpus siliculosus] | | |
| XLOC_006503 | ID_7639 | 12 | 0 | -0.42 | -0.1 | -2.26 | - | imm upregulated 3 [Ectocarpus siliculosus] | | |
| XLOC_013343 | ID_7955 | 12 | 0 | -0.51 | -0.29 | -1.83 | - | imm upregulated 3 [Ectocarpus siliculosus] | | |
|  |  |  |  |  |  |  |  |  |  |  |
|  |  |  |  |  |  |  |  |  |  |  |
|  |  |  |  |  |  |  |  |  |  |  |
|  |  |  |  |  |  |  |  |  |  |  |
|  |  |  |  |  |  |  |  |  |  |  |
