# Supplementary material for: Transcriptome sequencing of Saccharina japonica sporophytes during whole developmental periods reveals regulatory networks underlying alginate and mannitol biosynthesis
Source: BMC Genomics. 2019 Dec 12;20:975. doi: 10.1186/s12864-019-6366-x (PMC6909449; doi:10.1186/s12864-019-6366-x)
Supplement: Supplementary file 7 — Additional file 7: Figure S4. Contents of mannitol and alginate in S. japonica detected in different developmental stages and tissues from January to June. [file 12864_2019_6366_MOESM7_ESM.docx]

Content (%)

Fig. S4
