# Supplementary material for: Transcriptome sequencing of Saccharina japonica sporophytes during whole developmental periods reveals regulatory networks underlying alginate and mannitol biosynthesis
Source: BMC Genomics. 2019 Dec 12;20:975. doi: 10.1186/s12864-019-6366-x (PMC6909449; doi:10.1186/s12864-019-6366-x)
Supplement: Supplementary file 8 — Additional file 8: Table S4. Genes involved in the metabolism of alginate and mannitol in S. japonica. [file 12864_2019_6366_MOESM8_ESM.docx]

| Table S4 Genes involved in the metabolism of alginate and mannitol in *S. japonica* | | | | | |
| --- | --- | --- | --- | --- | --- |
| Name | Abbrev. | Gene ID | Module | ORF |  |
| mannose-6-phosphate isomerase | MPI1 | GENE_021848 | plum2 | complete |  |
|  | MPI2 | GENE_013980 | mediumpurple3 | complete |  |
|  | MPI3 | GENE_013986 | greenyellow | complete |  |
| phosphomannomutase | PMM1 | GENE_007314 | darkorange | complete |  |
|  | PMM2 | GENE_006655 | darkolivegreen | complete |  |
| GDP-mannose 6-dehydrogenase | GMD1 | GENE_022030 | greenyellow | complete |  |
|  | GMD2 | GENE_008524 | greenyellow | complete |  |
|  | GMD3 | GENE_022063 | darkorange | complete |  |
| mannuronan synthase | GT2 (MS) | GENE_006305 | greenyellow | complete |  |
| mannuronate C5-epimerase | MC5E1 | GENE_007233 | blue | complete |  |
|  | MC5E2 | GENE_019552 | blue | complete |  |
|  | MC5E3 | GENE_020029 | blue | complete |  |
|  | MC5E4 | GENE_012838 | darkolivegreen | partial |  |
|  | MC5E5 | GENE_012831 | maroon | complete |  |
|  | MC5E6 | GENE_012807 | blue | complete |  |
|  | MC5E7 | GENE_015182 | darkgreen | partial |  |
|  | MC5E8 | XLOC_010457 | black | partial |  |
|  | MC5E70 | GENE_007019 | mediumpurple3 | partial |  |
|  | MC5E122 | XLOC_006798 | mediumpurple3 | partial |  |
|  | … | … | … | … |  |
| mannitol 1-phosphate dehydrogenase | M1PDH1 | GENE _011959 | greenyellow | complete |  |
|  | M1PDH2 | GENE_003979 | plum2 | partial |  |
| mannitol-1-phosphatase | M1Pase | XLOC_010181 | saddlebrown | complete |  |
| mannitol dehydrogenase | M2DH | GENE_006978 | darkgreen | partial |  |
|  |  | GENE_006979 | darkgreen | partial |  |
| fructokinase | FK | GENE_018623 | darkolivegreen | complete |  |
|  |  |  |  |  |  |
|  |  |  |  |  |  |
