# Supplementary material for: Transcriptome sequencing of Saccharina japonica sporophytes during whole developmental periods reveals regulatory networks underlying alginate and mannitol biosynthesis
Source: BMC Genomics. 2019 Dec 12;20:975. doi: 10.1186/s12864-019-6366-x (PMC6909449; doi:10.1186/s12864-019-6366-x)
Supplement: Supplementary file 10 — Additional file 10: Table S5. The statistics of genes in each module. [file 12864_2019_6366_MOESM10_ESM.docx]

| Table S5 The statistics of genes in each module | | | |
| --- | --- | --- | --- |
| Module | No. of genes | percentage (%) |  |
| Darkolivegreen | 2798 | 16.67660031 |  |
| Greenyellow | 1827 | 10.88925974 |  |
| Blue | 1559 | 9.291929908 |  |
| Black | 1450 | 8.642269639 |  |
| Plum2 | 1364 | 8.129693646 |  |
| Darkgreen | 1229 | 7.325068542 |  |
| Tan | 1031 | 6.144951722 |  |
| Lightcyan | 941 | 5.608534986 |  |
| Corall | 728 | 4.339015377 |  |
| Darkorange2 | 618 | 3.683394922 |  |
| Darkorange | 589 | 3.510549529 |  |
| Lightgreen | 508 | 3.027774467 |  |
| Brown4 | 441 | 2.628442007 |  |
| Floralwhite | 354 | 2.109905829 |  |
| Darkslateblue | 345 | 2.056264155 |  |
| Saddlebrown | 250 | 1.490046489 |  |
| Violet | 220 | 1.311240911 |  |
| Mediumpurple3 | 193 | 1.15031589 |  |
| Lightpink4 | 114 | 0.679461199 |  |
| Maroon | 114 | 0.679461199 |  |
| Lavenderblush3 | 105 | 0.625819526 |  |
|  |  |  |  |
