# Supplementary material for: Transcriptome sequencing of Saccharina japonica sporophytes during whole developmental periods reveals regulatory networks underlying alginate and mannitol biosynthesis
Source: BMC Genomics. 2019 Dec 12;20:975. doi: 10.1186/s12864-019-6366-x (PMC6909449; doi:10.1186/s12864-019-6366-x)
Supplement: Supplementary file 12 — Additional file 12: Table S6. The enriched pathways in “brown4”, “darkgreen”, “black” and “darkslateblue” modules (p < 0.05). [file 12864_2019_6366_MOESM12_ESM.docx]

| Table S6 The enriched pathways in "brown4", "darkgreen", "black" and "darkslateblue" modules (p < 0.05) | | | | | | | |
| --- | --- | --- | --- | --- | --- | --- | --- |
| **Module** | **Pathway** | **DEGs genes with pathway annotation** | **All genes with pathway annotation** | **Pvalue** | **Qvalue** | **Pathway ID** |  |
| **Brown4** | Citrate cycle (TCA cycle) | 11 (15.94%) | 38 (1.43%) | 0 | 0 | ko00020 |  |
|  | Carbon metabolism | 16 (23.19%) | 165 (6.22%) | 0.000003 | 0.000089 | ko01200 |  |
|  | Microbial metabolism in diverse environments | 18 (26.09%) | 229 (8.64%) | 0.000012 | 0.000264 | ko01120 |  |
|  | Biosynthesis of antibiotics | 20 (28.99%) | 299 (11.28%) | 0.000039 | 0.000659 | ko01130 |  |
|  | Biosynthesis of secondary metabolites | 26 (37.68%) | 527 (19.88%) | 0.000395 | 0.005373 | ko01110 |  |
|  | Glyoxylate and dicarboxylate metabolism | 6 (8.7%) | 42 (1.58%) | 0.000625 | 0.007087 | ko00630 |  |
|  | Tropane, piperidine and pyridine alkaloid biosynthesis | 3 (4.35%) | 10 (0.38%) | 0.001777 | 0.017262 | ko00960 |  |
|  | Cysteine and methionine metabolism | 5 (7.25%) | 42 (1.58%) | 0.004158 | 0.03534 | ko00270 |  |
|  | Lysine degradation | 4 (5.8%) | 27 (1.02%) | 0.004695 | 0.035474 | ko00310 |  |
|  | Valine, leucine and isoleucine degradation | 5 (7.25%) | 47 (1.77%) | 0.00678 | 0.046107 | ko00280 |  |
|  | 2-Oxocarboxylic acid metabolism | 4 (5.8%) | 33 (1.24%) | 0.009736 | 0.060185 | ko01210 |  |
|  | Pyruvate metabolism | 5 (7.25%) | 59 (2.23%) | 0.017403 | 0.098616 | ko00620 |  |
|  | ABC transporters | 4 (5.8%) | 43 (1.62%) | 0.024176 | 0.126457 | ko02010 |  |
|  | Glycine, serine and threonine metabolism | 4 (5.8%) | 44 (1.66%) | 0.026083 | 0.126691 | ko00260 |  |
|  | Glycolysis / Gluconeogenesis | 5 (7.25%) | 72 (2.72%) | 0.037547 | 0.170214 | ko00010 |  |
|  | Biosynthesis of amino acids | 8 (11.59%) | 157 (5.92%) | 0.048515 | 0.197518 | ko01230 |  |
| **Darkgreen** | DNA replication | 7 (4.22%) | 35 (1.32%) | 0.005021 | 0.259817 | ko03030 |  |
|  | Regulation of autophagy | 8 (4.82%) | 49 (1.85%) | 0.009887 | 0.259817 | ko04140 |  |
|  | Arachidonic acid metabolism | 6 (3.61%) | 32 (1.21%) | 0.012768 | 0.259817 | ko00590 |  |
|  | Pyrimidine metabolism | 12 (7.23%) | 94 (3.55%) | 0.01283 | 0.259817 | ko00240 |  |
|  | Lipoic acid metabolism | 2 (1.2%) | 4 (0.15%) | 0.021507 | 0.329354 | ko00785 |  |
|  | Nucleotide excision repair | 7 (4.22%) | 48 (1.81%) | 0.027723 | 0.329354 | ko03420 |  |
|  | Ubiquitin mediated proteolysis | 11 (6.63%) | 93 (3.51%) | 0.028463 | 0.329354 | ko04120 |  |
| **Black** | Alanine, aspartate and glutamate metabolism | 7 (5.07%) | 33 (1.24%) | 0.001215 | 0.095987 | ko00250 |  |
|  | [Microbial metabolism in diverse environments](file:///D:\Programs\ProductionJournal\Temp\Table%20S1-S10%2020191105%20BMC.xlsx#RANGE!gene2) | 21 (15.22%) | 229 (8.64%) | 0.006352 | 0.250888 | ko01120 |  |
|  | [Valine, leucine and isoleucine degradation](file:///D:\Programs\ProductionJournal\Temp\Table%20S1-S10%2020191105%20BMC.xlsx#RANGE!gene3) | 7 (5.07%) | 47 (1.77%) | 0.009718 | 0.255903 | ko00280 |  |
|  | [Ubiquitin mediated proteolysis](file:///D:\Programs\ProductionJournal\Temp\Table%20S1-S10%2020191105%20BMC.xlsx#RANGE!gene4) | 10 (7.25%) | 93 (3.51%) | 0.020867 | 0.287436 | ko04120 |  |
|  | [Nitrogen metabolism](file:///D:\Programs\ProductionJournal\Temp\Table%20S1-S10%2020191105%20BMC.xlsx#RANGE!gene5) | 4 (2.9%) | 21 (0.79%) | 0.021062 | 0.287436 | ko00910 |  |
|  | [Carbon metabolism](file:///D:\Programs\ProductionJournal\Temp\Table%20S1-S10%2020191105%20BMC.xlsx#RANGE!gene6) | 15 (10.87%) | 165 (6.22%) | 0.022118 | 0.287436 | ko01200 |  |
|  | [Protein processing in endoplasmic reticulum](file:///D:\Programs\ProductionJournal\Temp\Table%20S1-S10%2020191105%20BMC.xlsx#RANGE!gene7) | 12 (8.7%) | 124 (4.68%) | 0.025469 | 0.287436 | ko04141 |  |
|  | [Glycolysis / Gluconeogenesis](file:///D:\Programs\ProductionJournal\Temp\Table%20S1-S10%2020191105%20BMC.xlsx#RANGE!gene8) | 8 (5.8%) | 72 (2.72%) | 0.031345 | 0.289011 | ko00010 |  |
|  | [Carbon fixation in photosynthetic organisms](file:///D:\Programs\ProductionJournal\Temp\Table%20S1-S10%2020191105%20BMC.xlsx#RANGE!gene9) | 6 (4.35%) | 47 (1.77%) | 0.032925 | 0.289011 | ko00710 |  |
|  | [Inositol phosphate metabolism](file:///D:\Programs\ProductionJournal\Temp\Table%20S1-S10%2020191105%20BMC.xlsx#RANGE!gene10) | 7 (5.07%) | 61 (2.3%) | 0.036851 | 0.291126 | ko00562 |  |
|  | [Citrate cycle (TCA cycle)](file:///D:\Programs\ProductionJournal\Temp\Table%20S1-S10%2020191105%20BMC.xlsx#RANGE!gene11) | 5 (3.62%) | 38 (1.43%) | 0.044697 | 0.321003 | ko00020 |  |
| **Darkslateblue** | Nitrogen metabolism | 2 (9.09%) | 21 (0.79%) | 0.012552 | 0.176877 | ko00910 |  |
|  | [Sulfur metabolism](file:///D:\Programs\ProductionJournal\Temp\Table%20S1-S10%2020191105%20BMC.xlsx#RANGE!gene2) | 2 (9.09%) | 24 (0.91%) | 0.01625 | 0.176877 | ko00920 |  |
|  | [Biosynthesis of secondary metabolites](file:///D:\Programs\ProductionJournal\Temp\Table%20S1-S10%2020191105%20BMC.xlsx#RANGE!gene3) | 9 (40.91%) | 527 (19.88%) | 0.018951 | 0.176877 | ko01110 |  |
|  | [Cysteine and methionine metabolism](file:///D:\Programs\ProductionJournal\Temp\Table%20S1-S10%2020191105%20BMC.xlsx#RANGE!gene4) | 2 (9.09%) | 42 (1.58%) | 0.04634 | 0.282305 | ko00270 |  |
|  |  |  |  |  |  |  |  |
|  |  |  |  |  |  |  |  |
