# Supplementary material for: Transcriptome sequencing of Saccharina japonica sporophytes during whole developmental periods reveals regulatory networks underlying alginate and mannitol biosynthesis
Source: BMC Genomics. 2019 Dec 12;20:975. doi: 10.1186/s12864-019-6366-x (PMC6909449; doi:10.1186/s12864-019-6366-x)
Supplement: Supplementary file 13 — Additional file 13: Table S7. The description of genes in enriched photosynthesis-relevant pathways which are highly correlated with greenyellow module (p < 0.05). [file 12864_2019_6366_MOESM13_ESM.docx]

| Table S7 The description of genes in enriched photosynthesis-relevant pathways which are highly correlated with greenyellow module (p < 0.05) | | | | | | |
| --- | --- | --- | --- | --- | --- | --- |
| GeneID | Connectivity | Symbol | Pathway | Annotation |  |  |
| XLOC_000589 | 171.5444848 | VHA-F | oxidative phosphorylation | vacuolar atp synthase subunit f [Nannochloropsis gaditana] |  |  |
| GENE_001658 | 148.8407146 | MTACP2 | oxidative phosphorylation | Acyl carrier protein [Ectocarpus siliculosus] |  |  |
| GENE_004675 | 164.4694273 | Atp6v1g2 | oxidative phosphorylation | H+-ATPase G subunit [Fragilariopsis cylindrus CCMP1102] |  |  |
| GENE_004797 | 140.5877945 | At2g33220 | oxidative phosphorylation | GRIM-19 [Coccomyxa subellipsoidea C-169] |  |  |
| GENE_005575 | 109.5054536 | Atp5c1 | oxidative phosphorylation | F-type H+-transporting ATPase subunit gamma [Nannochloropsis gaditana CCMP526] |  |  |
| GENE_007108 | 187.6774961 | vatD-1 | oxidative phosphorylation | ATPase, V0 complex, subunit D [Fragilariopsis cylindrus CCMP1102] |  |  |
| GENE_008100 | 191.5741595 | VAP | oxidative phosphorylation | V-type ATPase 16 kDa proteolipid subunit [Fragilariopsis cylindrus CCMP1102] |  |  |
| GENE_011294 | 146.9046257 | NDUFA9 | oxidative phosphorylation | NADH dehydrogenase (ubiquinone) [Ectocarpus siliculosus] |  |  |
| GENE_011383 | 100.0861276 | QCR7-2 | oxidative phosphorylation | ubiquinol cytochrome c reductase subunit QCR7 [Ectocarpus siliculosus] |  |  |
| GENE_012232 | 308.3345837 | vha-15 | oxidative phosphorylation | ARM repeat-containing protein [Fragilariopsis cylindrus CCMP1102] |  |  |
| GENE_013098 | 245.9711724 | VATE | oxidative phosphorylation | V-type proton ATPase subunit E [Blastocystis sp. ATCC 50177/Nand II] |  |  |
| GENE_019152 | 172.1635048 | - | oxidative phosphorylation | inorganic pyrophosphatase [Ectocarpus siliculosus] |  |  |
| GENE_019595 | 215.0576463 | NAD10 | oxidative phosphorylation | NUO10 homolog, NADH dehydrogenase (ubiquinone) subunit 10 [Ectocarpus siliculosus] |  |  |
| GENE_020559 | 212.6514258 | Oscp | oxidative phosphorylation | ATP synthase O subunit, mitochondrial precursor [Ectocarpus siliculosus] |  |  |
| GENE_022179 | 182.501458 | VAP | oxidative phosphorylation | V-type ATPase 16 kDa proteolipid subunit [Fragilariopsis cylindrus CCMP1102] |  |  |
| GENE_024356 | 149.5335328 | ATPC | oxidative phosphorylation | ATP synthase gamma chain [Ectocarpus siliculosus] |  |  |
| GENE_025103 | 33.01077016 | CYCL | oxidative phosphorylation | ubiquinol cytochrome c reductase cytochrome c1 prec (ISS) [Ectocarpus siliculosus] |  |  |
| GENE_025256 | 167.7562853 | VHA-c''2 | oxidative phosphorylation | v-type h-atpase subunit [Ectocarpus siliculosus] |  |  |
| GENE_025468 | 221.2742253 | Atp6v1b2 | oxidative phosphorylation | vacuolar ATP synthase subunit B [Ectocarpus siliculosus] |  |  |
| GENE_027932 | 147.6490238 | acpP | oxidative phosphorylation | Acyl carrier protein [Ectocarpus siliculosus] |  |  |
| GENE_028094 | 65.94216184 | CIB22 | oxidative phosphorylation | NADH dehydrogenase (ubiquinone) 1 beta subcomplex 9 [Blastocystis sp. ATCC 50177/Nand II] |  |  |
| GENE_028102 | 88.33259452 | - | oxidative phosphorylation | expressed unknown protein [Ectocarpus siliculosus] |  |  |
| GENE_005393 | 241.6785502 | CAB2 | Photosynthesis - antenna proteins | Light harvesting complex protein [Ectocarpus siliculosus] |  |  |
| GENE_006728 | 167.8982949 | LHCB4.2 | Photosynthesis - antenna proteins | Light harvesting complex protein [Ectocarpus siliculosus] |  |  |
| GENE_007838 | 275.8241985 | LHCA1 | Photosynthesis - antenna proteins | Light harvesting complex protein [Ectocarpus siliculosus] |  |  |
| GENE_010728 | 65.23165281 | L1818 | Photosynthesis - antenna proteins | Light harvesting complex protein [Ectocarpus siliculosus] |  |  |
| GENE_010760 | 42.29072188 | L1818 | Photosynthesis - antenna proteins | Light harvesting complex protein [Ectocarpus siliculosus] |  |  |
| GENE_012386 | 245.5942096 | FCPE | Photosynthesis - antenna proteins | Light harvesting complex protein [Ectocarpus siliculosus] |  |  |
| GENE_013351 | 250.1875193 | LHC | Photosynthesis - antenna proteins | Light harvesting complex protein [Ectocarpus siliculosus] |  |  |
| GENE_014821 | 159.9729883 | FCPB | Photosynthesis - antenna proteins | Chloroplast light harvesting protein lhcf5 [Saccharina japonica] |  |  |
| GENE_018804 | 172.0255707 | LHC | Photosynthesis - antenna proteins | Light harvesting complex protein [Ectocarpus siliculosus] |  |  |
| GENE_021338 | 274.8674802 | FCP | Photosynthesis - antenna proteins | Light harvesting complex protein [Ectocarpus siliculosus] |  |  |
| GENE_021344 | 248.9124772 | LHC | Photosynthesis - antenna proteins | Light harvesting complex protein [Ectocarpus siliculosus] |  |  |
| GENE_022155 | 210.3242889 | LHC | Photosynthesis - antenna proteins | Light harvesting complex protein [Ectocarpus siliculosus] |  |  |
| GENE_022269 | 267.5411789 | FCPF | Photosynthesis - antenna proteins | Light harvesting protein lhcf6 [Saccharina latissima] |  |  |
| GENE_022274 | 65.35985827 | FCPB | Photosynthesis - antenna proteins | Chloroplast light harvesting protein lhcf5 [Saccharina japonica] |  |  |
| GENE_024768 | 160.9132555 | FCPE | Photosynthesis - antenna proteins | Light harvesting complex protein [Ectocarpus siliculosus] |  |  |
| GENE_026489 | 239.0763559 | - | Photosynthesis - antenna proteins | Light harvesting complex protein [Ectocarpus siliculosus] |  |  |
| GENE_028555 | 291.4113657 | FCPB | Photosynthesis - antenna proteins | Light harvesting complex protein [Ectocarpus siliculosus] |  |  |
| GENE_028837 | 249.761222 | LHC | Photosynthesis - antenna proteins | Light harvesting complex protein [Ectocarpus siliculosus] |  |  |
| GENE_014910 | 254.1278482 | PsbU | photosynthesis | Photosystem II 12 kDa extrinsic protein [Ectocarpus siliculosus] |  |  |
| GENE_000180 | 258.3809577 | Os07g0147900 | photosynthesis | Ferredoxin-NADP oxidoreductase [Ectocarpus siliculosus] |  |  |
| GENE_001250 | 22.95152628 | FDX6 | photosynthesis | Ferredoxin [Nannochloropsis gaditana] |  |  |
| GENE_004674 | 174.3798552 | petF1 | photosynthesis | Phenylacetate- oxygenase subunit [Nannochloropsis gaditana] |  |  |
| GENE_005841 | 264.7638432 | PSBO | photosynthesis | Manganese stabilising protein [Ectocarpus siliculosus] |  |  |
| GENE_013537 | 156.4931154 | petC | photosynthesis | Cytochrome b6-f complex iron-sulfur subunit [Ectocarpus siliculosus] |  |  |
| GENE_014618 | 202.135796 | PSB27-1 | photosynthesis | Photosystem II 11 kDa protein [Ectocarpus siliculosus] |  |  |
| GENE_024356 | 149.5335328 | ATPC | photosynthesis | ATP synthase gamma chain [Ectocarpus siliculosus] |  |  |
| GENE_029199 | 231.2546959 | PPL1 | photosynthesis | Photosystem II oxygen evolution complex protein PsbP [Ectocarpus siliculosus] |  |  |
| GENE_003847 | 110.9498623 | CRYD | circadian rhythm-plant | cryptochrome 3 [Ectocarpus siliculosus] |  |  |
| GENE_017329 | 122.3716711 | cph1(PHYB) | circadian rhythm-plant | Phytochrome-like protein 3 [Ectocarpus siliculosus] |  |  |
| GENE_017333 | 50.31352739 | cph1(PHYB) | circadian rhythm-plant | Phytochrome-like protein 3 [Ectocarpus siliculosus] |  |  |
| GENE_005631 | 223.246518 | pks18(CHS) | circadian rhythm-plant | Polyketide Synthase III [Ectocarpus siliculosus] |  |  |
| GENE_027487 | 27.40623123 | CKB1(CK2beta) | circadian rhythm-plant | casein kinase 2 beta chain, CK2B [Fragilariopsis cylindrus CCMP1102] |  |  |
| GENE_021665 | 104.890616 | RFWD2(COP1) | circadian rhythm-plant | PREDICTED: E3 ubiquitin-protein ligase COP1 [Setaria italica] |  |  |
| GENE_000420 | 131.3684541 | crtQ(ZDS) | carotenoid biosynthesis | zeta-carotene desaturase, chloroplast precursor [Ectocarpus siliculosus] |  |  |
| GENE_000415 | 191.0740996 | crtQ(ZDS) | carotenoid biosynthesis | zeta-carotene desaturase, chloroplast precursor [Ectocarpus siliculosus] |  |  |
| GENE_021497 | 230.0904018 | LCY1(LCYB) | carotenoid biosynthesis | Lycopene beta cyclase, chloroplast precursor [Ectocarpus siliculosus] |  |  |
| GENE_027759 | 168.8342874 | CYP97B3(LUT5) | carotenoid biosynthesis | cytochrome P450 [Ectocarpus siliculosus] |  |  |
| GENE_027744 | 76.40075523 | CYP97A3(LUT5) | carotenoid biosynthesis | cytochrome P450 [Ectocarpus siliculosus] |  |  |
| GENE_017807 | 100.9331679 | VDE1 | carotenoid biosynthesis | violaxanthin de-epoxidase, chloroplast precursor [Ectocarpus siliculosus] |  |  |
| GENE_006795 | 68.07680499 | ZEP | carotenoid biosynthesis | Flavoprotein Monooxygenase [Ectocarpus siliculosus] |  |  |
| GENE_000054 | 268.467445 | RPI3 | carbon fixation in photosynthetic organisms | Ribose-5-phosphate isomerase [Ectocarpus siliculosus] |  |  |
| GENE_000154 | 241.9657614 | PGK | carbon fixation in photosynthetic organisms | phosphoglycerate kinase [Ectocarpus siliculosus] |  |  |
| GENE_002455 | 203.4904864 | Mdh2 | carbon fixation in photosynthetic organisms | malate dehydrogenase [Saccharina japonica] |  |  |
| GENE_004642 | 62.20878692 | fbp | carbon fixation in photosynthetic organisms | fructose 1,6-bisphosphatase [Ectocarpus siliculosus] |  |  |
| GENE_008411 | 168.895194 | FBP | carbon fixation in photosynthetic organisms | Fructose-bisphosphatase [Ectocarpus siliculosus] |  |  |
| GENE_013757 | 246.6926683 | tpiA | carbon fixation in photosynthetic organisms | triose-phosphate isomerase [Thalassiosira pseudonana CCMP1335] |  |  |
| GENE_014416 | 56.13253123 | pgk | carbon fixation in photosynthetic organisms | phosphoglycerate kinase [Ectocarpus siliculosus] |  |  |
| GENE_014503 | 132.5095715 | GPD | carbon fixation in photosynthetic organisms | glyceraldehyde-3-phosphate dehydrogenase [Saccharina latissima] |  |  |
| GENE_015257 | 156.4303419 | rpe | carbon fixation in photosynthetic organisms | ribulose-phosphate 3-epimerase [Ectocarpus siliculosus] |  |  |
| GENE_015279 | 235.7525709 | At1g32060 | carbon fixation in photosynthetic organisms | Phosphoribulokinase [Ectocarpus siliculosus] |  |  |
| GENE_017746 | 212.6932389 | GAPC1 | carbon fixation in photosynthetic organisms | chloroplast glyceraldehyde-3-phosphate dehydrogenase precursor [Saccharina latissima] |  |  |
| GENE_021763 | 177.9240489 | CSBP | carbon fixation in photosynthetic organisms | Sedoheptulose-bisphosphatase [Ectocarpus siliculosus] |  |  |
|  |  |  |  |  |  |  |
|  |  |  |  |  |  |  |
|  |  |  |  |  |  |  |
|  |  |  |  |  |  |  |
|  |  |  |  |  |  |  |
|  |  |  |  |  |  |  |
