# Supplementary material for: Transcriptome sequencing of Saccharina japonica sporophytes during whole developmental periods reveals regulatory networks underlying alginate and mannitol biosynthesis
Source: BMC Genomics. 2019 Dec 12;20:975. doi: 10.1186/s12864-019-6366-x (PMC6909449; doi:10.1186/s12864-019-6366-x)
Supplement: Supplementary file 14 — Additional file 14: Table S8. The transcription factors annotated in all the 22 modules. [file 12864_2019_6366_MOESM14_ESM.docx]

| Table S8 The transcription factors annotated in all the 22 modules | | | | |  |
| --- | --- | --- | --- | --- | --- |
| GeneID | Module | Connectivity | Symbol | Description |  |
| GENE_019144 | coral1 | 15.4732947 | DPBF3 | activating transcription factor 6 [Nannochloropsis gaditana] |  |
| XLOC_034737 | coral1 | 40.99179849 | - | activating transcription factor 6 [Nannochloropsis gaditana] |  |
| GENE_006427 | greenyellow | 156.0910896 | - | activating transcription factor 6 [Nannochloropsis gaditana] |  |
| GENE_015520 | greenyellow | 238.4198027 | nhp6 | ATHMG (ARABIDOPSIS THALIANA HIGH MOBILITY GROUP); transcription factor [Ectocarpus siliculosus] |  |
| GENE_002963 | darkgreen | 161.9729246 | - | C6 transcription factor Prf [Ectocarpus siliculosus] |  |
| GENE_008916 | darkgreen | 69.5690738 | MYB | c-myb-like transcription factor [Ectocarpus siliculosus] |  |
| GENE_005580 | darkgreen | 43.37887244 | GTF3C3 | general transcription factor 3C polypeptide 3-like isoform X4 [Ananas comosus] |  |
| XLOC_012937 | darkgreen | 146.3216149 | gtf2h2 | General transcription factor II H, polypeptide 2 [Ectocarpus siliculosus] |  |
| GENE_014075 | darkolivegreen | 74.82396691 | - | general transcription factor iih subunit, partial [Nannochloropsis gaditana] |  |
| GENE_012695 | violet | 3.830087061 | - | global transcription factor group [Ectocarpus siliculosus] |  |
| GENE_023741 | darkolivegreen | 199.2226448 | Hsf | Heat Shock transcription factor (Partial), partial [Ectocarpus siliculosus] |  |
| GENE_027724 | greenyellow | 76.35062403 | HSF3 | Heat Shock transcription factor (Partial), partial [Ectocarpus siliculosus] |  |
| GENE_021129 | blue | 184.3710352 | CAPS | Heat Shock transcription factor [Ectocarpus siliculosus] |  |
| XLOC_036222 | coral1 | 81.79671806 | Hsf1 | Heat Shock transcription factor [Ectocarpus siliculosus] |  |
| GENE_021126 | darkolivegreen | 36.23922433 | HSFB2C | Heat Shock transcription factor [Ectocarpus siliculosus] |  |
| GENE_011913 | greenyellow | 22.26945939 | HSFA4C | Heat Shock transcription factor [Ectocarpus siliculosus] |  |
| GENE_023779 | greenyellow | 153.1291108 | hsf1 | Heat Shock transcription factor [Ectocarpus siliculosus] |  |
| GENE_008366 | lightcyan | 28.70577149 | Hsf | Heat Shock transcription factor [Ectocarpus siliculosus] |  |
| GENE_024072 | plum2 | 10.71608321 | HSFA2B | Heat Shock transcription factor [Ectocarpus siliculosus] |  |
| GENE_001506 | darkolivegreen | 71.56349773 | NFYB3 | histone-like transcription factor [Ectocarpus siliculosus] |  |
| GENE_001494 | darkolivegreen | 167.9106234 | NFY2 | histone-like transcription factor [Ectocarpus siliculosus] |  |
| XLOC_031444 | darkorange | 42.43557747 | Chrac1 | histone-like transcription factor family (CBF/NF-Y) [Ectocarpus siliculosus] |  |
| XLOC_033549 | saddlebrown | 26.74221256 | NFYC4 | histone-like transcription factor family (CBF/NF-Y) [Ectocarpus siliculosus] |  |
| GENE_017366 | lightcyan | 21.22038303 | - | myb dna binding protein transcription factor-like protein [Nannochloropsis gaditana] |  |
| GENE_020637 | saddlebrown | 33.07180832 | - | MYB DNA binding protein/ transcription factor-like protein [Thalassiosira pseudonana CCMP1335] |  |
| GENE_025481 | black | 29.37697942 | MYBL2 | myb transcription factor [Nannochloropsis gaditana] |  |
| GENE_027443 | darkgreen | 96.51730147 | - | nf-x1-like transcription factor [Nannochloropsis gaditana] |  |
| GENE_004600 | plum2 | 188.6799616 | GTF2H4 | PREDICTED: RNA polymerase II transcription factor B subunit 2 [Vitis vinifera] |  |
| GENE_016577 | brown4 | 46.71137078 | brd4-a | PREDICTED: transcription factor GTE12 [Jatropha curcas] |  |
| GENE_009207 | darkgreen | 98.16215522 | - | Putative Del transcription factor (Partial), partial [Ectocarpus siliculosus] |  |
| XLOC_014040 | floralwhite | 17.40377589 | - | Putative heat Shock transcription factor [Ectocarpus siliculosus] |  |
| GENE_016934 | black | 93.23380794 | - | Putative NIN-like transcription factor [Ectocarpus siliculosus] |  |
| GENE_011480 | blue | 9.37504152 | - | Putative NIN-like transcription factor [Ectocarpus siliculosus] |  |
| XLOC_033699 | blue | 26.25829106 | - | Putative NIN-like transcription factor [Ectocarpus siliculosus] |  |
| GENE_010876 | darkolivegreen | 227.7879472 | - | Putative NIN-like transcription factor [Ectocarpus siliculosus] |  |
| GENE_003799 | greenyellow | 118.945289 | NLP2 | Putative NIN-like transcription factor [Ectocarpus siliculosus] |  |
| GENE_024306 | darkorange | 22.54364709 | rpa12 | RNA polymerase I transcription factor TFIIS subunit RPA12 [Klebsormidium flaccidum] |  |
| GENE_012559 | lightpink4 | 2.555664697 | bzpD | similar to activating transcription factor 6 [Ectocarpus siliculosus] |  |
| GENE_020826 | black | 11.38235809 | SBT6.1 | similar to membrane-bound transcription factor protease, site 1 [Ectocarpus siliculosus] |  |
| GENE_011505 | floralwhite | 55.71616483 | ZNF91 | Similar to metal response element-binding transcription factor-1 [Ectocarpus siliculosus] |  |
| GENE_008942 | black | 58.48152144 | BATDEDRAFT_20316 | Sir2-type regulatory transcription factor silent information regulator protein [Ectocarpus siliculosus] |  |
| XLOC_001509 | darkorange2 | 139.0796687 | BATDEDRAFT_20316 | Sir2-type regulatory transcription factor silent information regulator protein [Ectocarpus siliculosus] |  |
| XLOC_024470 | darkorange2 | 156.2616147 | - | Sir2-type regulatory transcription factor silent information regulator protein [Ectocarpus siliculosus] |  |
| XLOC_005549 | darkgreen | 37.91599339 | TFB5 | TFIIH basal transcription factor complex TTD-A subunit [Galdieria sulphuraria] |  |
| GENE_009937 | black | 34.21307703 | - | Transcription factor 25 [Nannochloropsis gaditana] |  |
| GENE_005535 | darkolivegreen | 36.63801098 | Tfdp1 | transcription factor dp-1, partial [Chrysochromulina sp. CCMP291] |  |
| GENE_008289 | black | 57.39302858 | - | transcription factor E2F [Ectocarpus siliculosus] |  |
| XLOC_030807 | black | 92.4898002 | Hid1 | Transcription factor IIB [Klebsormidium flaccidum] |  |
| GENE_027227 | floralwhite | 40.88161562 | gtf2e1-1 | transcription factor iie [Nannochloropsis gaditana] |  |
| GENE_011367 | darkgreen | 40.04505575 | lid | transcription factor jumonji [Klebsormidium flaccidum] |  |
| GENE_024724 | darkolivegreen | 185.6399875 | - | transcription factor protein [Ectocarpus siliculosus] |  |
| GENE_024727 | darkolivegreen | 217.203409 | - | transcription factor protein [Ectocarpus siliculosus] |  |
| GENE_017020 | plum2 | 59.54353054 | HSF2 | Transcription factor prr1 (Pombe response regulator 1) [Ectocarpus siliculosus] |  |
| GENE_001864 | saddlebrown | 16.44694888 | GTF2H3 | transcription factor Tfb4 [Coccomyxa subellipsoidea C-169] |  |
| GENE_010521 | plum2 | 79.41376261 | TCX6 | TSO1; transcription factor (Partial) [Ectocarpus siliculosus] |  |
| GENE_006848 | greenyellow | 139.9630272 | - | zinc finger-containing transcription factor, putative [Ectocarpus siliculosus] |  |
| GENE_006855 | greenyellow | 154.3805259 | aflR | zinc finger-containing transcription factor, putative [Ectocarpus siliculosus] |  |
|  |  |  |  |  |  |
