# Supplementary material for: Transcriptome sequencing of Saccharina japonica sporophytes during whole developmental periods reveals regulatory networks underlying alginate and mannitol biosynthesis
Source: BMC Genomics. 2019 Dec 12;20:975. doi: 10.1186/s12864-019-6366-x (PMC6909449; doi:10.1186/s12864-019-6366-x)
Supplement: Supplementary file 15 — Additional file 15: Table S9. The transcription factors correlated with mannitol and alginate biosynthesis. [file 12864_2019_6366_MOESM15_ESM.docx]

| Table S9 The transcription factors correlated with mannitol and alginate biosynthesis | | | | |  |
| --- | --- | --- | --- | --- | --- |
| GeneID | Module | Connectivity | Symbol | Description |  |
| GENE_000097 | black | 46.629426 | MBF1B | flagellar associated protein, transcriptional coactivator-like protein [Ectocarpus siliculosus] |  |
| GENE_027078 | black | 138.05373 | - | Mitochodrial transcription termination factor [Nannochloropsis gaditana] |  |
| GENE_025481 | black | 29.376979 | MYBL2 | myb transcription factor [Nannochloropsis gaditana] |  |
| GENE_016934 | black | 93.233808 | - | Putative NIN-like transcription factor [Ectocarpus siliculosus] |  |
| GENE_020826 | black | 11.382358 | SBT6.1 | similar to membrane-bound transcription factor protease, site 1 [Ectocarpus siliculosus] |  |
| GENE_008942 | black | 58.481521 | BATDEDRAFT_20316 | Sir2-type regulatory transcription factor silent information regulator protein [Ectocarpus siliculosus] |  |
| GENE_009937 | black | 34.213077 | - | Transcription factor 25 [Nannochloropsis gaditana] |  |
| GENE_008289 | black | 57.393029 | - | transcription factor E2F [Ectocarpus siliculosus] |  |
| XLOC_030807 | black | 92.4898 | Hid1 | Transcription factor IIB [Klebsormidium flaccidum] |  |
| GENE_015511 | brown4 | 23.866752 | CNOT4 | CCR4-NOT transcription complex subunit 4 [Klebsormidium flaccidum] |  |
| XLOC_031444 | darkorange | 42.435577 | Chrac1 | histone-like transcription factor family (CBF/NF-Y) [Ectocarpus siliculosus] |  |
| GENE_024306 | darkorange | 22.543647 | rpa12 | RNA polymerase I transcription factor TFIIS subunit RPA12 [Klebsormidium flaccidum] |  |
| GENE_006427 | greenyellow | 156.09109 | - | activating transcription factor 6 [Nannochloropsis gaditana] |  |
| GENE_015520 | greenyellow | 238.4198 | nhp6 | ATHMG (ARABIDOPSIS THALIANA HIGH MOBILITY GROUP); transcription factor [Ectocarpus siliculosus] |  |
| GENE_027724 | greenyellow | 76.350624 | HSF3 | Heat Shock transcription factor (Partial), partial [Ectocarpus siliculosus] |  |
| GENE_023779 | greenyellow | 153.12911 | hsf1 | Heat Shock transcription factor [Ectocarpus siliculosus] |  |
| GENE_011913 | greenyellow | 22.269459 | HSFA4C | Heat Shock transcription factor [Ectocarpus siliculosus] |  |
| GENE_003799 | greenyellow | 118.94529 | NLP2 | Putative NIN-like transcription factor [Ectocarpus siliculosus] |  |
| GENE_006855 | greenyellow | 154.38053 | aflR | zinc finger-containing transcription factor, putative [Ectocarpus siliculosus] |  |
| GENE_006848 | greenyellow | 139.96303 | - | zinc finger-containing transcription factor, putative [Ectocarpus siliculosus] |  |
|  |  |  |  |  |  |
