# Supplementary material for: Transcriptome sequencing of Saccharina japonica sporophytes during whole developmental periods reveals regulatory networks underlying alginate and mannitol biosynthesis
Source: BMC Genomics. 2019 Dec 12;20:975. doi: 10.1186/s12864-019-6366-x (PMC6909449; doi:10.1186/s12864-019-6366-x)
Supplement: Supplementary file 16 — Additional file 16: Table S10. Primers of alginate and mannitol relevant genes used for RT-qPCR verification. [file 12864_2019_6366_MOESM16_ESM.docx]

| Table S10 Primers of alginate and mannitol relevant genes used for RT-qPCR verification | | | | |
| --- | --- | --- | --- | --- |
| **Gene name** | **Gene ID** | **Primers** | **Sequences (5’ to 3’)** |  |
| *MPI2* | GENE_013980 | qMPI-F | AGACGACAGCAGTTACGACG |  |
|  |  | qMPI-R | GTCGGTATGGCGTTGCTTTC |  |
| *PMM1* | GENE_007314 | qPMM-F | ATCAGAGAGACGATGGTTGCG |  |
|  |  | qPMM-R | TACTCGGCGGTGCCCTCTC |  |
| *GMD3* | GENE_022063 | qGMD-F | TCGAGAAGATGAACGTCGCC |  |
|  |  | qGMD-R | GGATGACGAAGATAGGCGGG |  |
| *MC5E70* | GENE_007019 | qMC5E70-F | GCCGTCGTCAAAGGGAACTA |  |
|  |  | qMC5E70-R | GAGGAGCCGGTGACTTTGTT |  |
| *MC5E122* | XLOC_006798 | qMC5E122-F | AGGCGAAGAAGAACATGGG |  |
|  |  | qMC5E122-R | GGTTAGGCCGTAGCTCTCAC |  |
| *M1PDH1* | GENE_011959 | qM1PDH1-F | TGACGCTAAGCTGCAAAGGA |  |
|  |  | qM1PDH1-R | CCGTAGATGTAGGTGCGGTC |  |
| *M1Pase* | XLOC_010181 | qM1Pase-F | GCAACAGCGTTTGTATCCCC |  |
|  |  | qM1Pase-R | CATGGCGTTGTTCACGTTGT |  |
| *M2DH* | GENE_006979 | qM2DH-F | GCGAGGCAGGACACTGAAGACC |  |
|  |  | qM2DH-R | GGGACCACATCCAGCACCAAC |  |
| *FK* | GENE_018623 | qFK-F | CTGTACTGTACTCCGACCGC |  |
|  |  | qFK-R | TCATTACGTAGGGCTTCGGC |  |
| *β-Actin* | *-* | qActin-F | GACGGGTAAGGAAGAACGG |  |
|  |  | qActin-R | GGGACAACCAAAACAAGGGCAGGAT |  |
|  |  |  |  |  |
